# Supplementary material for: Induced pluripotent stem cell-derived macrophages enable broad modeling of human inflammasome signaling
Source: Cell Rep Methods. 2026 Jun 25;6(8):101506. doi: 10.1016/j.crmeth.2026.101506 (PMC13494535; doi:10.1016/j.crmeth.2026.101506)
Supplement: Supplementary file 1 [file mmc1.pdf]

Cell Reports Methods, Volume *6*

Supplemental information

Induced pluripotent stem cell-derived macrophages enable broad modeling of human inflammasome signaling

Chloe M. McKee, Melanie Cranston, Emma C. McKay, Mohammad Arefian, Thea J. Mawhinney, Ben C. Collins, and Rebecca C. Coll

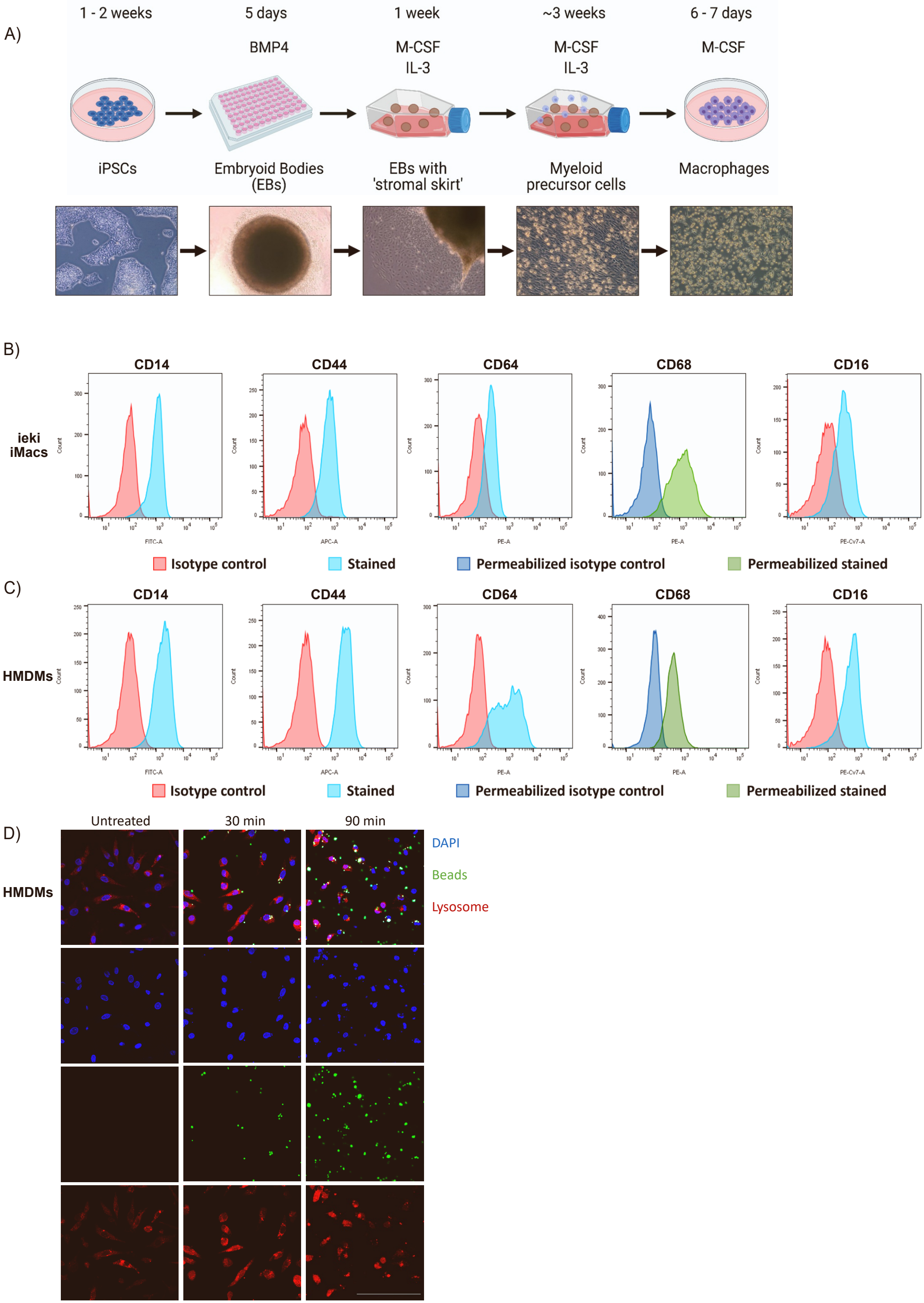

A)
1 - 2 weeks
5 days
1 week
~3 weeks
6 - 7 days
BMP4
M-CSF
IL-3
M-CSF
IL-3
M-CSF
iPSCs
Embryoid Bodies (EBs)
EBs with 'stromal skirt'
Myeloid precursor cells
Macrophages
B)
ieki iMacs
CD14
CD44
CD64
CD68
CD16
Count
FITC-A
APC-A
PE-A
PE-A
PE-Cy7-A
Isotype control
Stained
Permeabilized isotype control
Permeabilized stained
C)
HMDMs
CD14
CD44
CD64
CD68
CD16
Isotype control
Stained
Permeabilized isotype control
Permeabilized stained
D)
HMDMs
Untreated
30 min
90 min
DAPI
Beads
Lysosome

Fig. S1 HMDMs express macrophage markers and are highly phagocytic, related to Fig. 1.

A) Diagram illustrating the iMac differentiation protocol. Expression of macrophage markers in **B)** ieki_2 iMacs and **C)** HMDMs compared to relative isotype controls. **D)** Confocal microscope images from HMDMs following incubation with fluorescent beads. Scale bar = 100 μM.

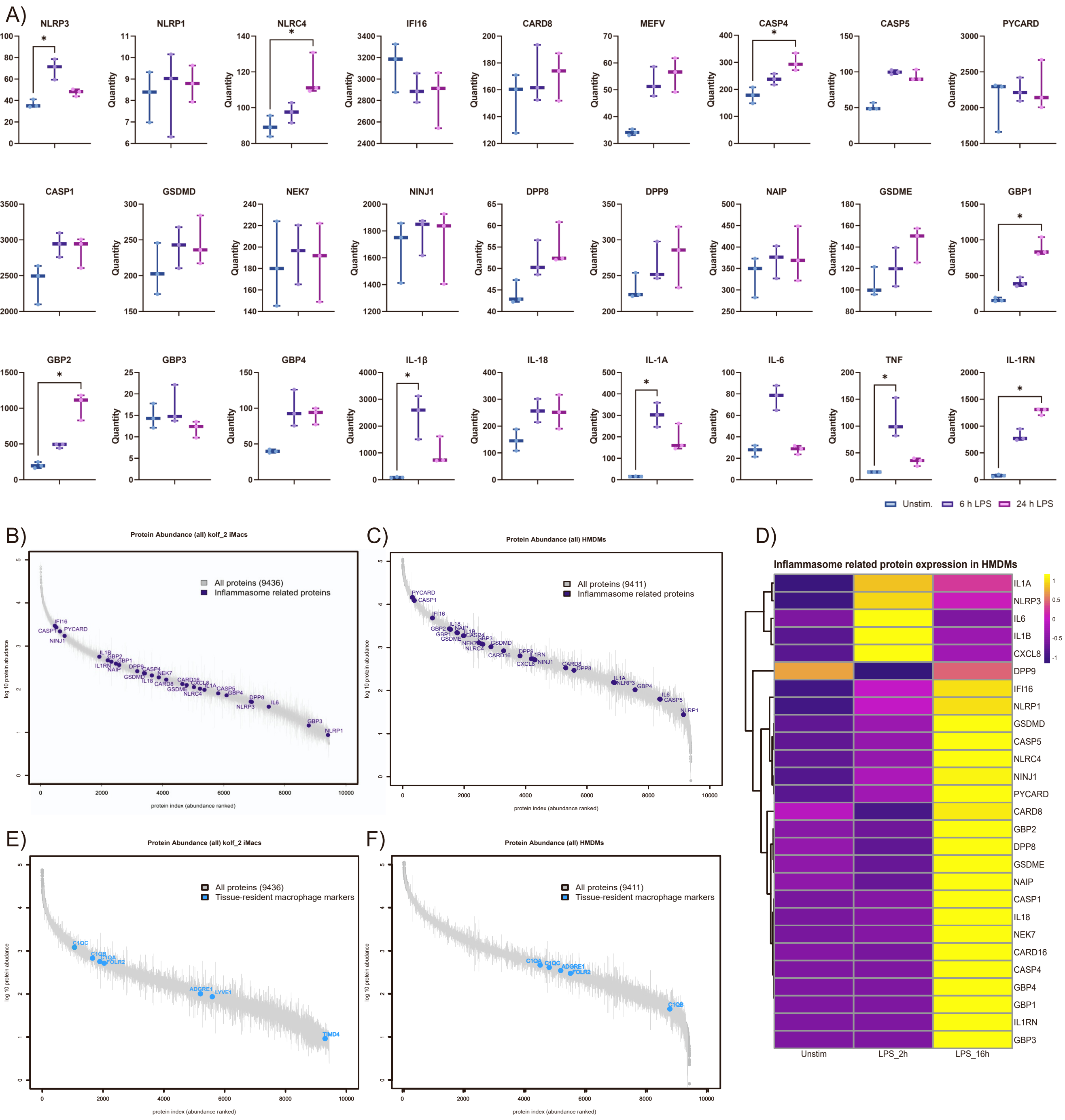
A)
NLRP3
NLRP1
NLRC4
IFI16
CARD8
MEFV
CASP4
CASP5
PYCARD
CASP1
GSDMD
NEK7
NINJ1
DPP8
DPP9
NAIP
GSDME
GBP1
GBP2
GBP3
GBP4
IL-1β
IL-18
IL-1A
IL-6
TNF
IL-1RN
Quantity
Unstim.
6 h LPS
24 h LPS
B)
Protein Abundance (all) kolf_2 iMacs
All proteins (9436)
Inflammasome related proteins
C)
Protein Abundance (all) HMDMs
All proteins (9411)
Inflammasome related proteins
D)
Inflammasome related protein expression in HMDMs
IL1A
NLRP3
IL6
IL1B
CXCL8
DPP9
IFI16
NLRP1
GSDMD
CASP5
NLRC4
NINJ1
PYCARD
CARD8
GBP2
DPP8
GSDME
NAIP
CASP1
IL18
NEK7
CARD16
CASP4
GBP4
GBP1
IL1RN
GBP3
Unstim
LPS_2h
LPS_16h
E)
Protein Abundance (all) kolf_2 iMacs
All proteins (9436)
Tissue-resident macrophage markers
C1QC
C1QB
C1QA
FOLR2
ADGRE1
LYVE1
TIMD4
F)
Protein Abundance (all) HMDMs
All proteins (9411)
Tissue-resident macrophage markers
log 10 protein abudance
protein index (abundance ranked)

Fig. S2 Expression of inflammasome-related proteins is similar between iMacs and HMDMs, related to Fig. 1. A) Expression of inflammasome- and inflammation-related proteins in kolf_2 iMacs following 6h/24h LPS. Data represented as mean +/- SEM, n = 3 replicates, *p< 0.05 by Two-way ANOVA with Šídák's multiple comparisons. Ranked average Log10 protein abundance of inflammasome-related proteins indicated in **B)** kolf_2 iMacs **C)** HMDMs. **D)** Protein quantity heatmap of inflammasome- and inflammation-related proteins following global proteomics analysis of HMDMs. Ranked average Log10 protein abundance of tissue-resident macrophage-associated proteins in **E)** kolf_2 iMacs and **F)** HMDMs. n = 3 kolf_2 iMac replicates, n = 4 buffy coat donors.

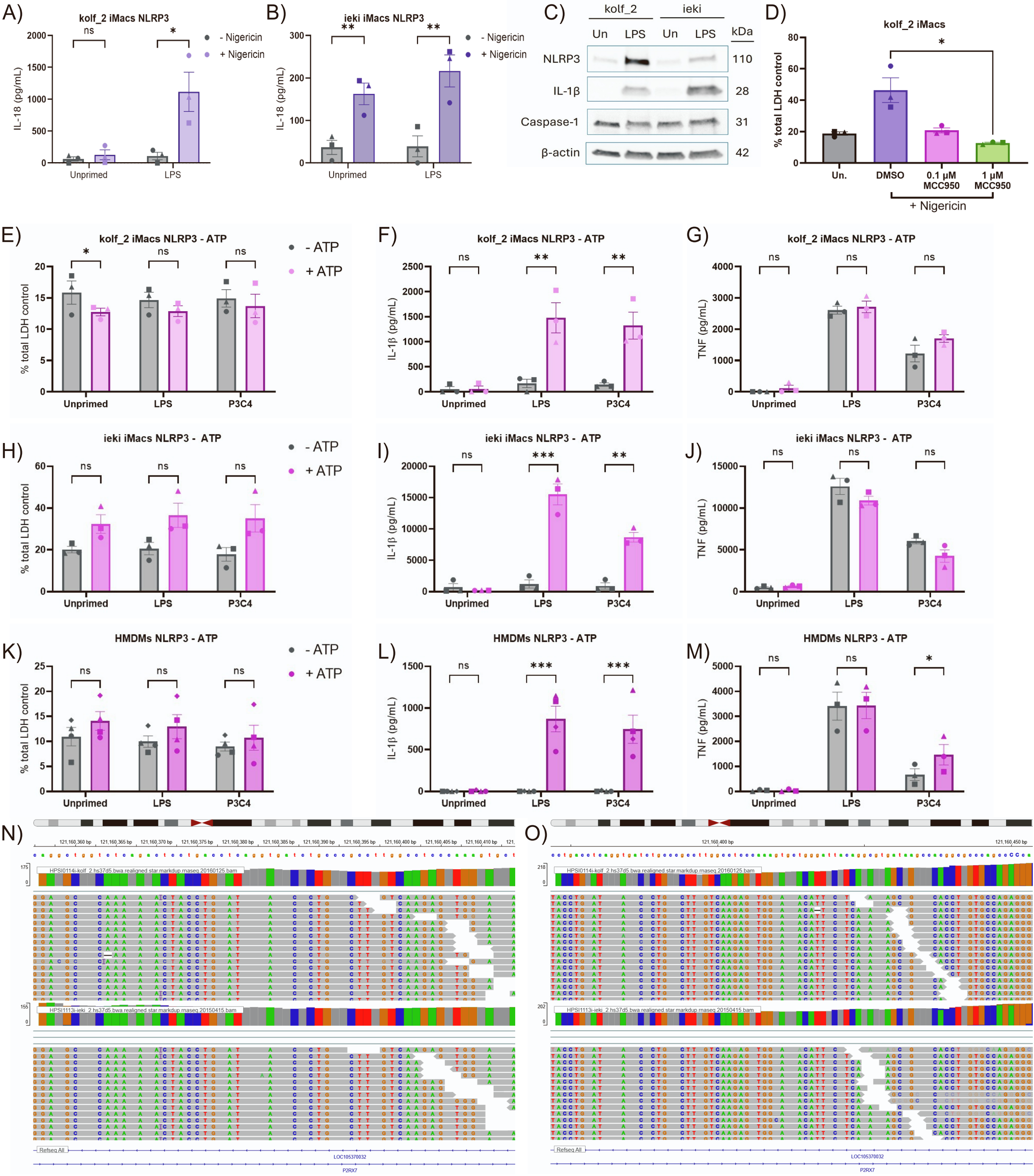

A)
kolf_2 iMacs NLRP3
IL-18 (pg/mL)
- Nigericin
+ Nigericin
Unprimed
LPS
ns
*
B)
ieki iMacs NLRP3
IL-18 (pg/mL)
- Nigericin
+ Nigericin
Unprimed
LPS
**
**
C)
kolf_2
ieki
Un
LPS
Un
LPS
kDa
NLRP3
110
IL-1β
28
Caspase-1
31
β-actin
42
D)
kolf_2 iMacs
% total LDH control
Un.
DMSO
0.1 µM MCC950
1 µM MCC950
+ Nigericin
*
E)
kolf_2 iMacs NLRP3 - ATP
% total LDH control
- ATP
+ ATP
Unprimed
LPS
P3C4
F)
kolf_2 iMacs NLRP3 - ATP
IL-1β (pg/mL)
Unprimed
LPS
P3C4
G)
kolf_2 iMacs NLRP3 - ATP
TNF (pg/mL)
Unprimed
LPS
P3C4
H)
ieki iMacs NLRP3 - ATP
% total LDH control
- ATP
+ ATP
Unprimed
LPS
P3C4
I)
ieki iMacs NLRP3 - ATP
IL-1β (pg/mL)
Unprimed
LPS
P3C4
J)
ieki iMacs NLRP3 - ATP
TNF (pg/mL)
Unprimed
LPS
P3C4
K)
HMDMs NLRP3 - ATP
% total LDH control
- ATP
+ ATP
Unprimed
LPS
P3C4
L)
HMDMs NLRP3 - ATP
IL-1β (pg/mL)
Unprimed
LPS
P3C4
M)
HMDMs NLRP3 - ATP
TNF (pg/mL)
Unprimed
LPS
P3C4
N)
HPSI0114i-kolf_2.hs37d5.bwa.realigned.star.markdup.rnaseq.20160125.bam
HPSI1113i-ieki_2.hs37d5.bwa.realigned.star.markdup.rnaseq.20150415.bam
Refseq All
LOC105370032
P2RX7
O)
HPSI0114i-kolf_2.hs37d5.bwa.realigned.star.markdup.rnaseq.20160125.bam
HPSI1113i-ieki_2.hs37d5.bwa.realigned.star.markdup.rnaseq.20150415.bam
Refseq All
LOC105370032
P2RX7

Fig. S3 NLRP3 activation in response to ATP is broadly comparable in iMacs and HMDMs, related to Fig. 2. IL-18 release from **A)** kolf_2 and **B)** ieki_2 iMacs following 4h LPS and 2h nigericin. **C)** Western blot from unstimulated and 6h LPS-primed kolf_2 and ieki_2 iMacs. **D)** LDH release from kolf_2 iMacs pre-treated with MCC950 (30 min) before nigericin (2h). LDH, IL-1β, and TNF release from **E-G)** kolf_2 iMacs, **H-J)** ieki_2 iMacs, and **K-M)** HMDMs following 4h LPS/Pam3CSK4 and 2h ATP. Data represented as mean +/- SEM, n = 3 iMac replicates, n = 4 buffy coat donors, *p< 0.05, **p< 0.01, ***p< 0.001, ****p< 0.0001 by Two-way ANOVA with Šídák's multiple comparisons. **N)** and **O)** Representative IGV screenshots showing read alignments (BAM files) for kolf_2 iPSCs (top) and ieki_2 iPSCs (bottom) indicating mismatches (transparent), insertions (lilac), and deletions (-) between the two cell lines.

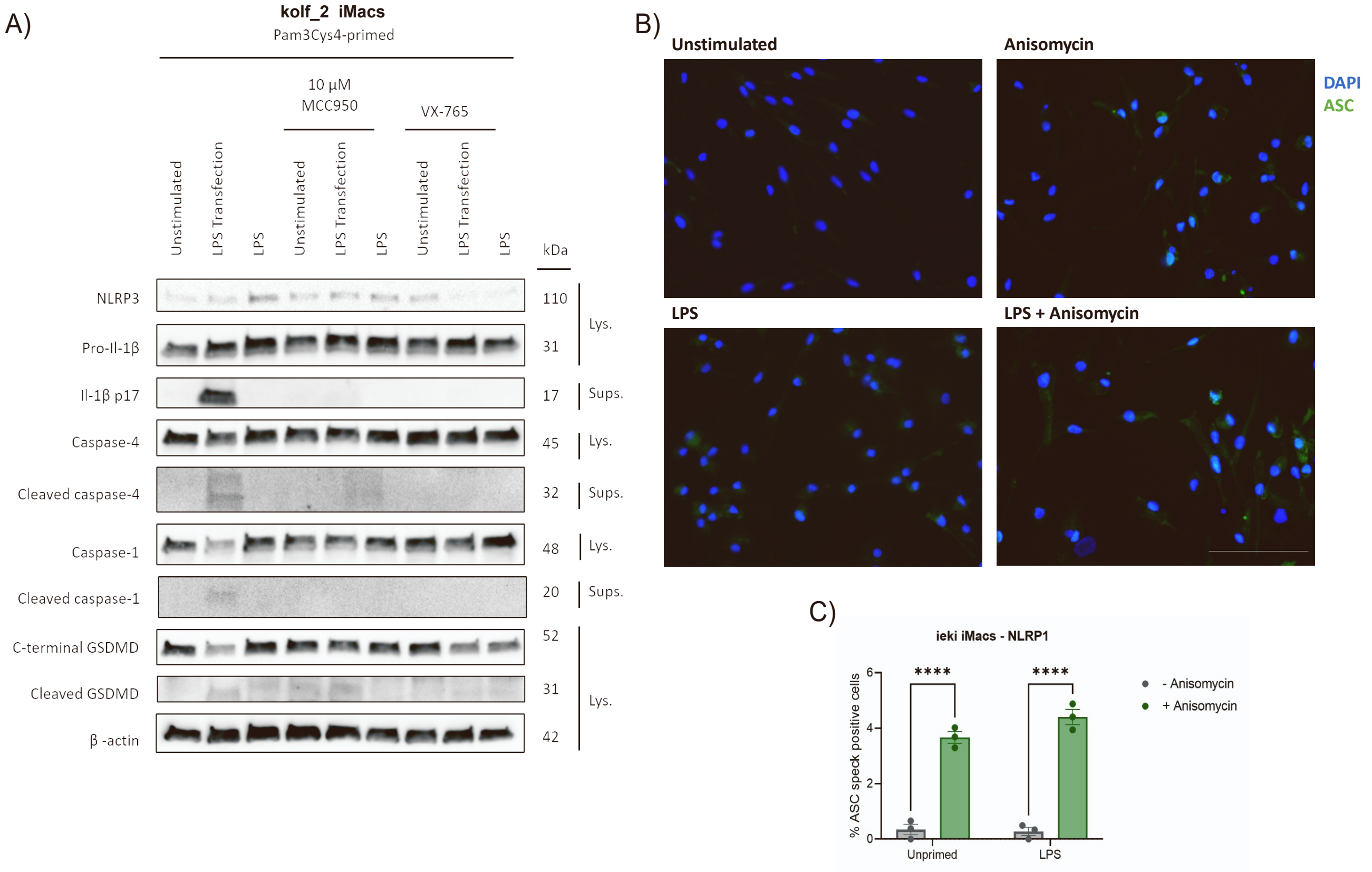

Fig. S4 NCI activation and NLRP1-induced ASC speck formation in iMacs, related to Fig. 3 and Fig. 4. A) Western blot from 16h Pam3CSK4-primed kolf_2 iMacs following 5h LPS transfection or LPS stimulation and pre-treatment with MCC950 or VX-765 (30 min). **B)** Fluorescent images from ieki_2 iMacs following 4h LPS and 24h anisomycin. Scale bar = 100 µM. **C)** Quantification of ASC speck positive cells. Data represented as mean +/- SEM, n = 3 iMac ieki_2 replicates, *p< 0.05, ***p< 0.001, ****p< 0.0001 by repeated measures ANOVA with Tukey's multiple comparisons test.

Donor	Date	Blood Type	Sex	Haemochromatosis (Y/N)	Figure No
1	27.10.20	N/A	N/A	N/A	4D-F
2	10.11.20	N/A	N/A	N/A	4D-F
3	26.11.20	N/A	N/A	N/A	4D-F
4	13.01.21	N/A	N/A	N/A	4D-F
5	26.01.21	N/A	N/A	N/A	4D-F
6	02.06.21	N/A	N/A	N/A	2 G-I, S4K-M
7	15.06.21	N/A	N/A	N/A	2 G-I, S4K-M
8	20.07.21	N/A	N/A	N/A	2 G-I, S4K-M
9	03.08.21	N/A	N/A	N/A	2 G-I, S4K-M
10	13.01.23	B+	N/A	N	S3B,C,E
11	13.01.23	O-	N/A	N	S3B,C,E
12	20.01.23	O+	N/A	N	S3B,C,E
13	20.01.23	O+	N/A	N	S3B,C,E
25	01.02.24	O-	F	N	1C, 3I-K, S1B
26	01.02.24	A	F	N	1C & F, 3I-K, S1B & D
27	15.02.24	O+	F	N	1C, 3I-K, S1B
28	15.02.24	A+	F	N	1C, 3I-K, S1B
31	22.03.25	O+	M	N	1F, S1D
32	22.03.25	O+	F	N	1F, S1D
39	29.07.24	A-	F	N	3D, E
49	17.1.25	A-	M	N	3D, E
50	17.1.25	A+	F	N	3D, E
51	27.11.25	A+	F	N	4J-L
52	05.02.26	O-	F	N	4J-L
53	05.02.26	B+	F	N	4J-L

Table S2 Buffy coat characteristics, related to STAR Methods.
