# Supplementary material for: Induced pluripotent stem cell-derived macrophages enable broad modeling of human inflammasome signaling
Source: Cell Rep Methods. 2026 Jun 25;6(8):101506. doi: 10.1016/j.crmeth.2026.101506 (PMC13494535; doi:10.1016/j.crmeth.2026.101506)
Supplement: Supplementary file 2 [file mmc3.pdf]

Cell Reports Methods

Report

Induced pluripotent stem cell-derived macrophages enable broad modeling of human inflammasome signaling

Graphical abstract

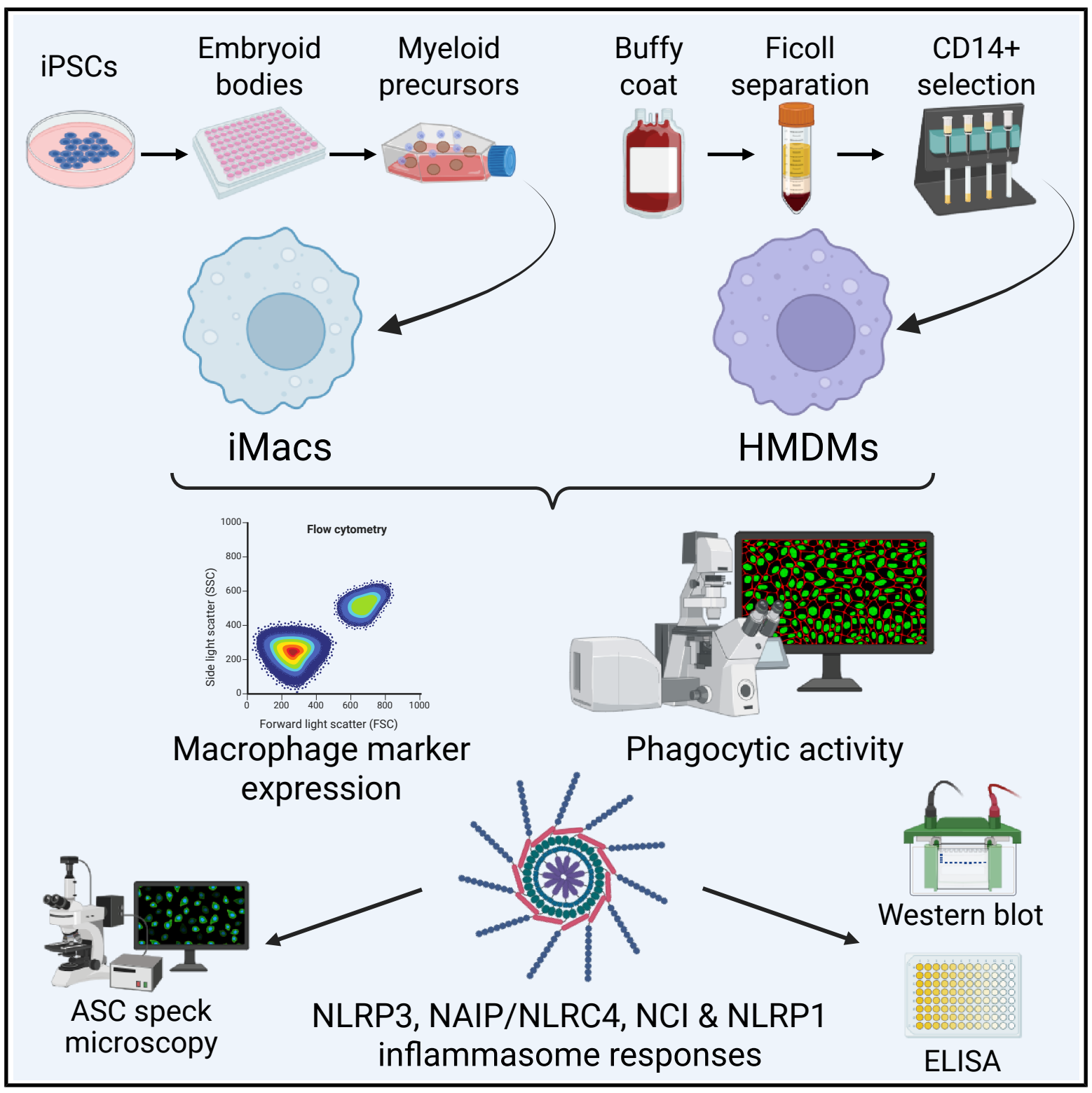

Highlights

- iPSC-derived macrophages enable broad interrogation of human inflammasomes
- iMacs express macrophage markers and inflammasome proteins and are phagocytotic
- iMacs are highly responsive to multiple inflammasome priming and activation stimuli
- iMacs are broadly comparable to monocyte-derived macrophages for inflammasome studies

Authors

Chloe M. McKee, Melanie Cranston, Emma C. McKay, Mohammad Arefian, Thea J. Mawhinney, Ben C. Collins, Rebecca C. Coll

Correspondence

r.coll@qub.ac.uk

In brief

Current *in vitro* macrophage models have limitations for studying inflammasome function. McKee et al. demonstrate that iPSC-derived macrophages are broadly comparable to monocyte-derived macrophages in response to a wide range of inflammasome priming and activation stimuli and thus hold promise as a physiologically relevant model for human inflammasome studies.

McKee et al., 2026, Cell Reports Methods *6*, 101506
August 17, 2026 © 2026 The Authors. Published by Elsevier Inc.
https://doi.org/10.1016/j.crmeth.2026.101506

Cell Reports Methods

CellPress
OPEN ACCESS

Report

Induced pluripotent stem cell-derived macrophages enable broad modeling of human inflammasome signaling

Chloe M. McKee,[1] Melanie Cranston,[1] Emma C. McKay,[1,2] Mohammad Arefian,[2] Thea J. Mawhinney,[1] Ben C. Collins,[2] and Rebecca C. Coll[1,3,*]
[1]Wellcome-Wolfson Institute for Experimental Medicine, Queen's University of Belfast, Belfast, UK
[2]School of Biological Sciences, Queen's University of Belfast, Belfast, UK
[3]Lead contact
*Correspondence: r.coll@qub.ac.uk
https://doi.org/10.1016/j.crmeth.2026.101506

MOTIVATION iPSC-derived macrophages (iMacs) are functionally, transcriptionally, and phenotypically similar to primary human macrophages. iMacs, therefore, offer new opportunities to study inflammasome activity in a human macrophage model, but the properties of inflammasome biology in iMacs and their fidelity to primary human cells have not been well established. In this study, we describe a protocol to differentiate and characterize iMacs. We describe how to activate a range of inflammasomes within iMacs and assess inflammasome responses. We also benchmark iMacs against the gold-standard human monocyte-derived macrophage model.

SUMMARY

Macrophage models are a mainstay of inflammasome research; however, current *in vitro* models have significant limitations. Here, we generate induced pluripotent stem cell (iPSC)-derived macrophages (iMacs) to study inflammasome signaling and benchmark them with human monocyte-derived macrophages. We confirm that iMacs express high levels of macrophage markers and are highly phagocytic. Proteomic analysis shows iMacs express many inflammasome sensors and related proteins, and iMacs respond to multiple inflammasome stimuli. The NLRP3 inflammasome is strongly activated in iMacs, and nigericin alone activates NLRP3. The non-canonical inflammasome does not require a priming step, and high NAIP/NLRC4 activation is observed in response to needle toxin. Finally, unlike human monocyte-derived macrophages (HMDMs), talabostat activates NLRP1 in iMacs. Therefore, we demonstrate that iMacs are a physiologically relevant and attractive model to study inflammasome signaling.

INTRODUCTION

Macrophages are key innate immune cells that express a wide range of pattern recognition receptors (PRRs). Cytosolic PRRs such as nucleotide oligomerization domain (NOD)-like receptors (NLRs) can assemble into inflammasomes.[1] Inflammasomes are activated by many microbial-, host-, and environmental-derived stimuli to trigger potent inflammatory responses.[2] Inflammasome activation induces recruitment of apoptosis-associated speck-like protein containing a CARD (ASC). ASC filaments provide a platform to activate caspase-1, which cleaves pro-inflammatory cytokines interleukin (IL)-1β and IL-18 and causes gasdermin D (GSDMD)-mediated cell death (pyroptosis).[2] Aberrant inflammasome activation is associated with many inflammatory diseases, and inflammasome inhibitors are of significant therapeutic interest. Indeed, several NOD-, LRR-, and pyrin domain-containing protein 3 (NLRP3) inflammasome inhibitors are in clinical trials for conditions including asthma and Parkinson's disease.[3,4] Inflammasome receptors, such as NLRP3 and NLR family CARD domain-containing protein 4 (NLRC4), are expressed primarily by myeloid cells.[5] However, current *in vitro* macrophage models have significant limitations.

Primary mouse bone marrow-derived macrophages are widely used, but significant differences exist in the immune systems between mice and humans.[6] Importantly, there are significant differences in the expression, structure, and activation of inflammasomes. Seven NLRP1 paralogs and seven NAIPs that trigger activation of NLRC4 are expressed in mice, while humans express a single NLRP1 gene and a single functional NAIP.[7–9] In addition, regulatory proteins such as caspase recruitment domain (CARD)-only and pyrin domain

This is an open access article under the CC BY license (http://creativecommons.org/licenses/by/4.0/).

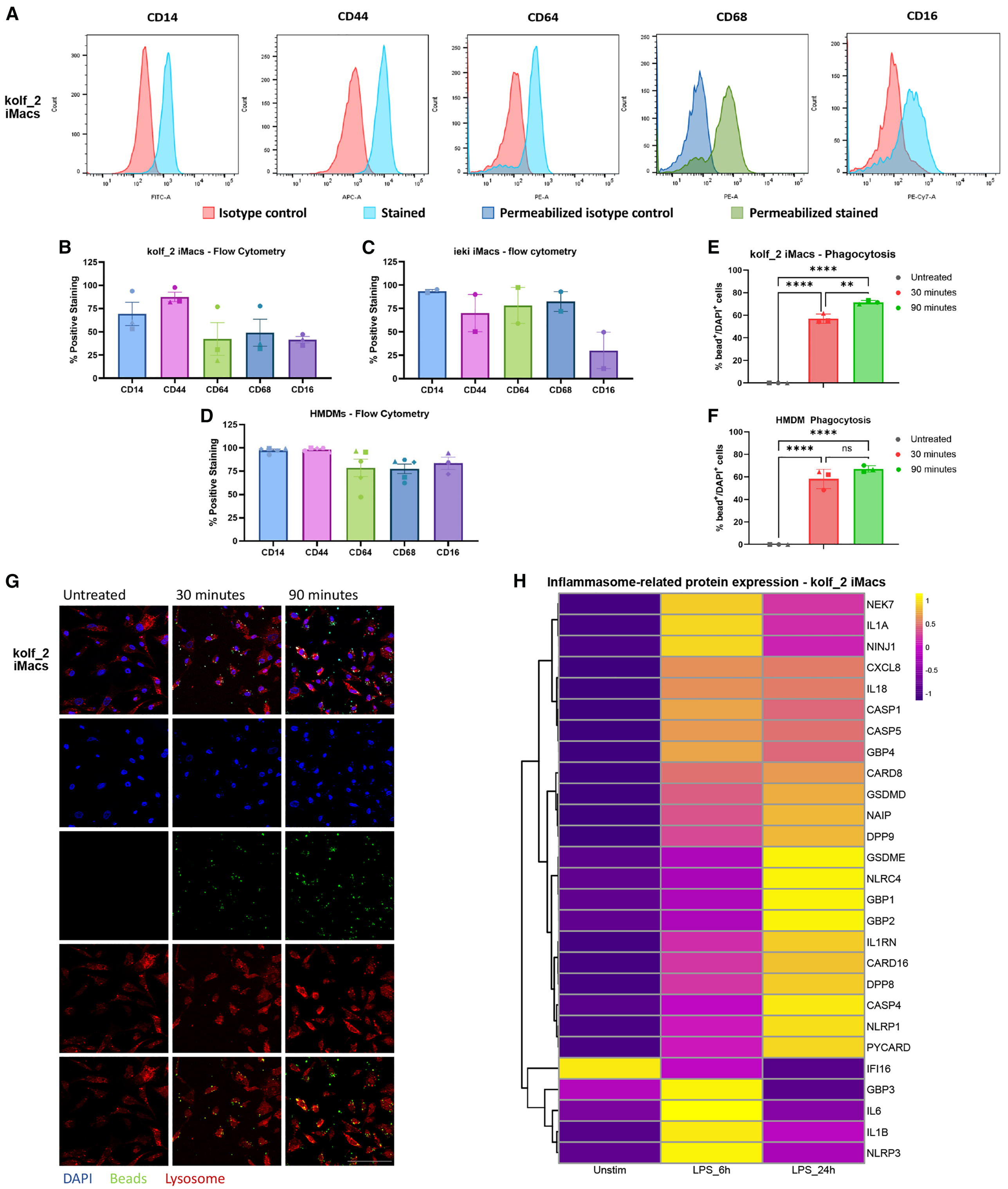

Figure 1. iMacs are macrophage like with high expression of macrophage markers, inflammasome components, and phagocytosis activity similar to HMDMs

(A–E) (A) Expression of macrophage markers in kolf_2 iMacs compared to isotype controls. Macrophage marker expression in (B) kolf_2 iMacs, *n* = 3 differentiation batches; (C) ieki_2 iMacs, *n* = 2 differentiation batches; and (D) HMDMs, *n* = 5 donors. Quantification of DAPI+/bead+ cells in (E) kolf_2

(legend continued on next page)

(PYD)-only proteins (COPs/POPs) are not expressed in mice.[10] Interestingly, we recently identified small-molecule NLRP3 inhibitors, BAL-0028 and BAL-0598, that are highly effective in human cells but have significantly reduced activity in mice.[11] To explore the clinical benefits of targeting inflammasomes, it is critical to understand inflammasome regulation in human systems.

Current human models include the human monocytic leukemia THP-1 cell line, which can be differentiated into a macrophage-like state using phorbol-12-myristate-13-acetate (PMA).[12] However, THP-1s are functionally immature and have karyotypic abnormalities, and PMA differentiation increases pro-IL-1β and NLRP3 expression, confounding inflammasome assay interpretation.[13–15] Human monocyte-derived macrophages (HMDMs) are the current gold standard.[16] However, HMDMs are non-proliferative and difficult to genetically modify, and experimental responses can vary significantly due to the inherent heterogenic differences between donors.[14,16,17] In addition, HMDMs are not representative of tissue-resident macrophages as they have different embryological origins.[18] Therefore, new *in vitro* human macrophage models overcoming these limitations would be useful for studying inflammasomes.

Human induced pluripotent stem cells (iPSCs) are a powerful tool to model human development and disease across many cell types, including macrophages. Various protocols have been developed for differentiating iPSCs into macrophages (iPSC-derived macrophages [iMacs]), including embryoid body (EB) formation.[19–22] Recent protocols form EBs by centrifugation with bone morphogenetic protein 4 (BMP-4).[23] EB-independent protocols have also recently been published that require additional growth factors/supplements compared with EB-dependent protocols.[23,24] iMacs express a wide range of macrophage-specific markers and display conserved responses to inflammatory stimuli, high levels of phagocytosis, and antimicrobial activity.[16,17,25] iMac differentiation protocols offer a continuous, scalable supply of cells that can be genotype and patient specific.[16,22,26] iPSCs are amenable to genetic manipulation and can be modified before differentiation into macrophages.[16] iMacs, therefore, offer new opportunities to study macrophage biology.

To date, few published studies investigate inflammasome responses in iMacs. Staphylococcal pore-forming toxins activate NLRP3 in iMacs,[27] and iMacs differentiated from iPSCs derived from a patient with neonatal-onset multisystem inflammatory disease display constitutive NLRP3 activation.[28] Most recently, iMacs have been shown to activate NLRP3 independently of NIMA-related kinase 7 (NEK7).[29] However, there has been no in-depth characterization of other inflammasome responses within iMacs. Further investigation was, therefore, required to determine whether iMacs are comparable to HMDMs for inflammasome studies.

RESULTS

Expression of macrophage markers

To generate iMacs, we used the Douthwaite et al.[23] protocol, adapting the number of iPSCs used to form EBs and using large flasks to bulk up precursor yield and increase sterility with filtered caps for extended culture. This protocol is summarized in Figure S1A. We used kolf_2 iPSCs, a skin-derived cell line generated from a healthy male of European ancestry. Kolf_2 iPSCs are widely used in the stem cell field and maintain genomic integrity during gene editing.[23] We also used ieki_2 iPSCs, a skin-derived cell line generated from a healthy female of European ancestry to demonstrate that our results were more broadly applicable.[30]

To confirm our protocol generates macrophage-like cells, we examined macrophage marker expression by flow cytometry. While monocytes express most of these markers, CD16 expression is low in monocytes (∼10%).[23] CD14 is highly expressed in kolf_2 and ieki_2 iMacs (Figures 1A–1C and S1B) comparable to HMDMs (Figures S1C and 1D). A small $CD14^{-}$ population is present in kolf_2 iMacs (Figure 1B). This has previously been characterized as $CD45^{+}CD34^{-}$, indicating they are of hematopoietic origin and are no longer stem cell progenitors.[31] CD44, CD64, and CD68 are highly expressed, although expression varies between iMac batches and HMDM donors (Figures 1B–1D). CD16 expression in iMacs is lower than the other markers (Figures 1B and 1C) in line with previous findings.[22,23] In contrast, HMDMs express higher levels of CD16 (Figure 1D). Therefore, iMacs express a range of macrophage markers, indicating they are macrophage like.

Phagocytic activity

Macrophages play vital roles in phagocytosing microbes, releasing pro-inflammatory cytokines, and presenting antigens to activate adaptive immune responses.[32] To characterize their functionality, we assessed phagocytosis of fluorescent beads. iMacs are highly phagocytic, with ∼55% of cells containing at least one fluorescent bead after 30 min and ∼70% of cells after 90 min (Figures 1E and 1G). This is highly consistent with HMDMs (Figures 1F and S1D).

Expression of inflammasome-related proteins

Inflammasome sensor expression varies between cell types; for example, airway epithelial cells basally express low levels of NLRP3 but high NLRP1.[33] Previous studies have not examined global protein expression in iMacs; therefore, we performed proteomic analysis on kolf_2 iMacs following lipopolysaccharide (LPS) stimulation (Table S1). iMacs express inflammasome sensors including NLRP3, NLRP1, CARD8, NAIP, NLRC4, and caspase-4/5 alongside inflammasome components including ASC, caspase-1, GSDMD, NINJ1, and NEK7 (Figure 1H). In agreement with previous studies,[34] NLRP3 expression is enhanced after 6-h LPS, but decreases after 24 h (Figures 1H and S2A). NLRC4 and

iMacs, $n = 3$, and (F) HMDMs donors, $n = 3$. Data represented as mean ± SEM. $^{**}p < 0.01$ and $^{****}p < 0.0001$ by one-way ANOVA with Tukey's multiple comparisons.

(G) Confocal microscopic images from kolf_2 iMacs incubated with fluorescent beads. Scale bar, 100 μM.

(H) Scaled protein quantity heatmap of inflammasome- and inflammation-related proteins following whole-cell proteomics analysis of kolf_2 iMacs stimulated with LPS, $n = 3$.

caspase-4 also increase with LPS stimulation and peak at 24 h (Figures 1H and S2A). iMacs respond to 6-h LPS by increasing the expression of pro-IL-1β and other inflammasome-independent cytokines such as IL-6 (Figures 1H and S2A). In a similar dataset from HMDMs treated with LPS for 2 or 16 h, the overall expression of inflammasome-related proteins is broadly comparable with kolf_2 iMacs, with ASC (PYCARD), interferon gamma-inducible protein 16 (IFI16), and caspase-1 among the top expressed proteins (Figures S2B and S2C). In addition, the pattern of protein expression in response to LPS is similar, with pro-IL-1β, IL-6, and NLRP3 peaking at early time points, while NLRC4, NLRP1, caspase-4, and GSDME peak later (Figures 1H and S2D). These data demonstrate that iMacs express inflammasome sensors and pathway components and regulate expression in response to LPS comparably to HMDMs. Additionally, tissue-resident macrophage markers including TIMD4 and LYVE1 are detected in iMacs but not HMDMs, while C1QA, C1QB, C1QC, and FOLR2[35] have higher relative expression in iMacs (Figures S2E and S2F). Therefore, these proteomic datasets suggest that iMacs are more tissue-resident-like.

Response to NLRP3 stimuli

To functionally characterize iMacs as a human inflammasome signaling model, we first investigated NLRP3 activation. In macrophages, NLRP3 activation requires a priming stimulus followed by an activation signal.[36] To activate NLRP3, iMacs were primed with LPS (Toll-like receptor 4, TLR4) or Pam3CSK4 (TLR1/2) and activated with the bacterial toxin nigericin or danger signal extracellular ATP. As expected, nigericin triggers pyroptosis in primed kolf_2 and ieki_2 iMacs, alongside high IL-1β release and lower but significant IL-18 release (Figures 2A, 2B, 2D, 2E, S3A, and S3B). IL-1β release was higher in ieki_2 iMacs (Figures 2B and 2E), which may be due to elevated pro-IL-1β expression (Figure S3C). In response to priming, there is also significant tumor necrosis factor (TNF) release from both cell lines (Figures 2C and 2F), an inflammasome-independent cytokine induced following TLR stimulation. Priming also enhances pro-IL-1β and NLRP3 expression in kolf_2 iMacs (Figure 2J). In HMDMs, priming plus nigericin triggers NLRP3 activation to a lower level than iMacs, with little pyroptosis and lower IL-1β release, whereas TNF release is comparable with kolf_2 iMacs (Figures 2G–2I). Interestingly, significant pyroptosis is seen in unprimed iMacs treated with nigericin, but not in HMDMs (Figures 2A, 2D, and 2G). Caspase-1 and GSDMD cleavage in kolf_2 iMacs stimulated with nigericin alone is similar to primed cells (Figure 2J), indicating this is inflammasome-dependent pyroptosis, although there is no corresponding IL-18 release in kolf_2 iMacs (Figure S3A). Meanwhile in ieki_2 iMacs, there is significant IL-18 in response to nigericin alone (Figure S3B). Treatment with the NLRP3-specific inhibitor MCC950 also blocks nigericin alone-induced pyroptosis (Figure S3D), confirming nigericin alone activates NLRP3 in iMacs.

The response to ATP in kolf_2 iMacs is lower than nigericin, with no increase in pyroptosis and lower IL-1β release (Figures S3E–S3G). In ieki_2 iMacs, the response to ATP is higher, with a non-significant increase in pyroptosis and similar IL-1β release as the nigericin-treated cells (Figures S3H–S3J). ATP triggers IL-1β release in HMDMs without pyroptosis, comparable with kolf_2 iMacs (Figures S3K–S3M). The different levels of response to ATP in kolf_2 and ieki_2 iMacs could be explained by polymorphisms in the ATP receptor (P2X7R) gene that are present in the human population.[37] Indeed, analysis of the P2X7R gene in publicly available RNA datasets for kolf_2 and ieki_2 cell lines shows mismatches, insertions, and deletions between the two cell lines (Figures S3N and S3O).

To confirm NLRP3 inflammasome formation in iMacs, ASC specks were visualized. Priming followed by nigericin stimulation triggers a significant increase in DAPI+ASC+ iMacs (Figures 2K and 2L). In unprimed cells treated with nigericin, there is a non-significant increase in DAPI+ASC+ cells (Figure 2L), further confirming that nigericin alone can induce NLRP3 activation. Together, these data demonstrate that NLRP3 is functional in iMacs and responsive to multiple priming and activation stimuli.

Response to non-canonical inflammasome stimulation

We next examined non-canonical inflammasome (NCI) activation. In human cells, the NCI is activated by cytosolic LPS, which triggers caspase-4/5 activation, pyroptosis, and IL-18 release.[38] GSDMD pores indirectly activate NLRP3 via potassium efflux, activating caspase-1 and causing IL-1β release.[39] To study NCI activation, Pam3CSK4-primed kolf_2 iMacs were transfected with LPS. An extracellular LPS control was included to confirm responses are due to cytosolic LPS. LPS transfection causes increased pyroptosis, IL-1β release, and caspase-1, caspase-4, and GSDMD cleavage in unprimed iMacs (Figures 3A–3C). This is blocked by the caspase-1/4 inhibitor VX-765. MCC950 partially reduces IL-1β release and caspase-1 cleavage in LPS-transfected cells, but does not affect pyroptosis or caspase-4 cleavage (Figures 3A–3C), consistent with previous findings in HMDMs.[39]

All responses are similar in Pam3CSK4-primed iMacs but there is increased IL-1β release due to enhanced pro-IL-1β (Figures 3A, 3B, and S4A). In unprimed iMacs, increased pro-IL-1β and NLRP3 expression is observed both in cells transfected with LPS or in cells treated with extracellular LPS (Figure 3C). This may explain why IL-1β release is observed from unprimed cells as transfected LPS can also prime iMacs within the 5-h transfection.

The response to NCI activation in HMDMs is similar to iMacs as LPS transfection increases pyroptosis and IL-1β release, which is inhibited with VX-765 (Figures 3D and 3E). However, priming with Pam3CSK4 is more necessary in HMDMs as IL-1β release in unprimed LPS-transfected cells is lower than in iMacs (Figures 3B, 3E), indicating that iMacs are more sensitive to LPS.

Response to NAIP/NLRC4 stimulation

In humans, NAIP detects components of bacterial type III secretion systems and associates with NLRC4 to trigger inflammasome formation.[40] For NAIP/NLRC4 inflammasome activation, kolf_2 iMacs were primed and treated with Lfn-BsaL, plus anthrax protective antigen (PA). Lfn-BsaL plus PA alone induces significant pyroptosis (Figure 3F). Priming does not enhance Lfn-BsaL-induced pyroptosis, but in primed cells, Lfn-BsaL induces significant IL-1β release (Figure 3G). TNF release is also induced in response to priming (Figure 3H). The responses in iMacs are very similar to those observed in HMDMs, with similar levels of

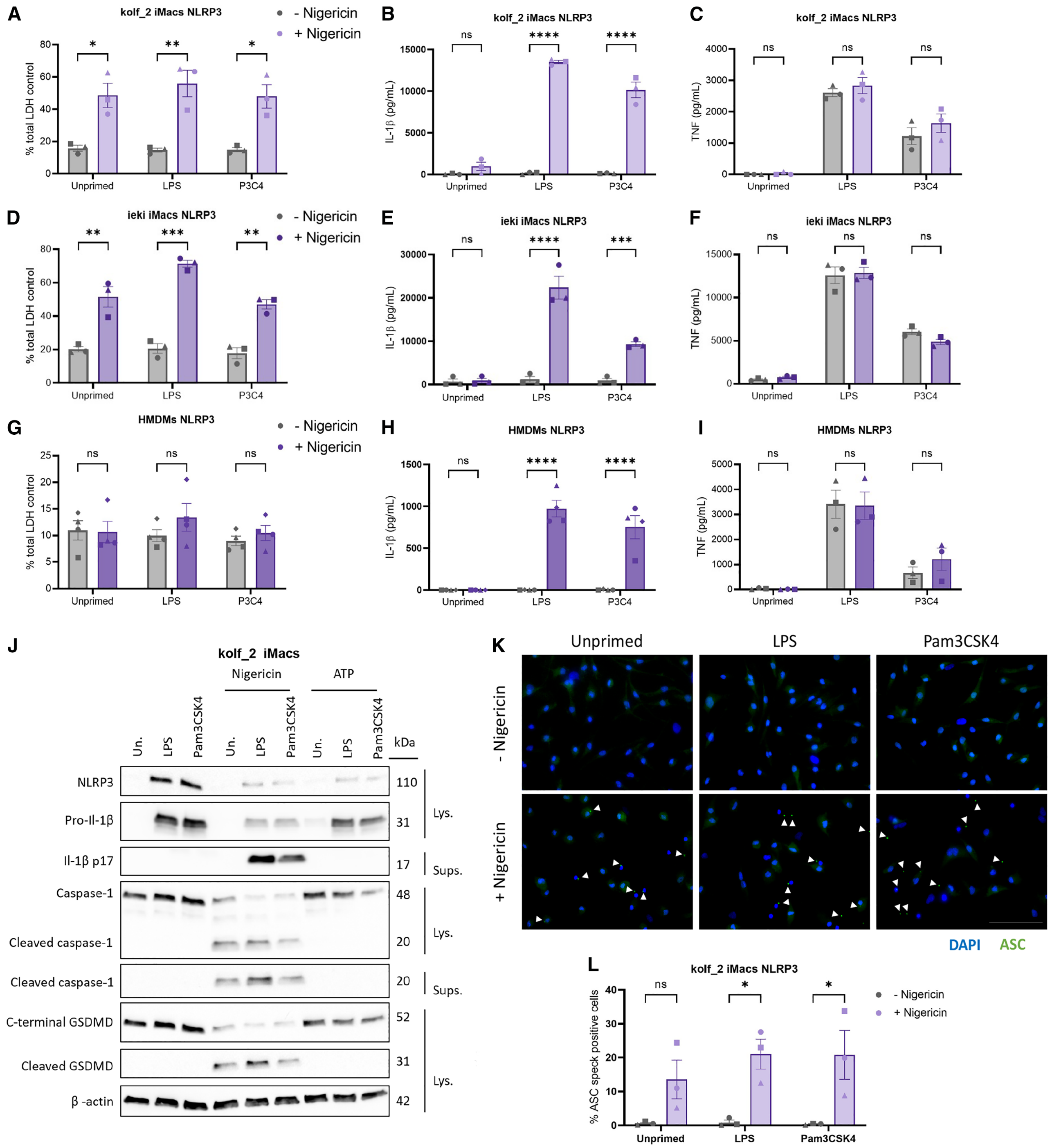

Figure 2. iMacs display higher NLRP3 responses compared with HMDMs

(A–J) LDH, IL-1β, and TNF release from (A–C) kolf_2 iMacs, (D–F) ieki_2 iMacs, and (G–I) HMDMs, and (J) Western blot from kolf_2 iMacs following 4-h LPS/Pam3CSK4 and 2-h nigericin.

(K) Images from kolf_2 iMacs following stimulation as above. Scale bar, 100 μM.

(L) Quantification of DAPI+/speck+ cells.

Data represented as mean ± SEM, *n* = 3 kolf_2/ieki_2 iMac replicates and *n* = 4 HMDM donors, **p* < 0.05, ***p* < 0.01, ****p* < 0.001, and *****p* < 0.0001 by two-way ANOVA with Šídák's multiple comparisons.

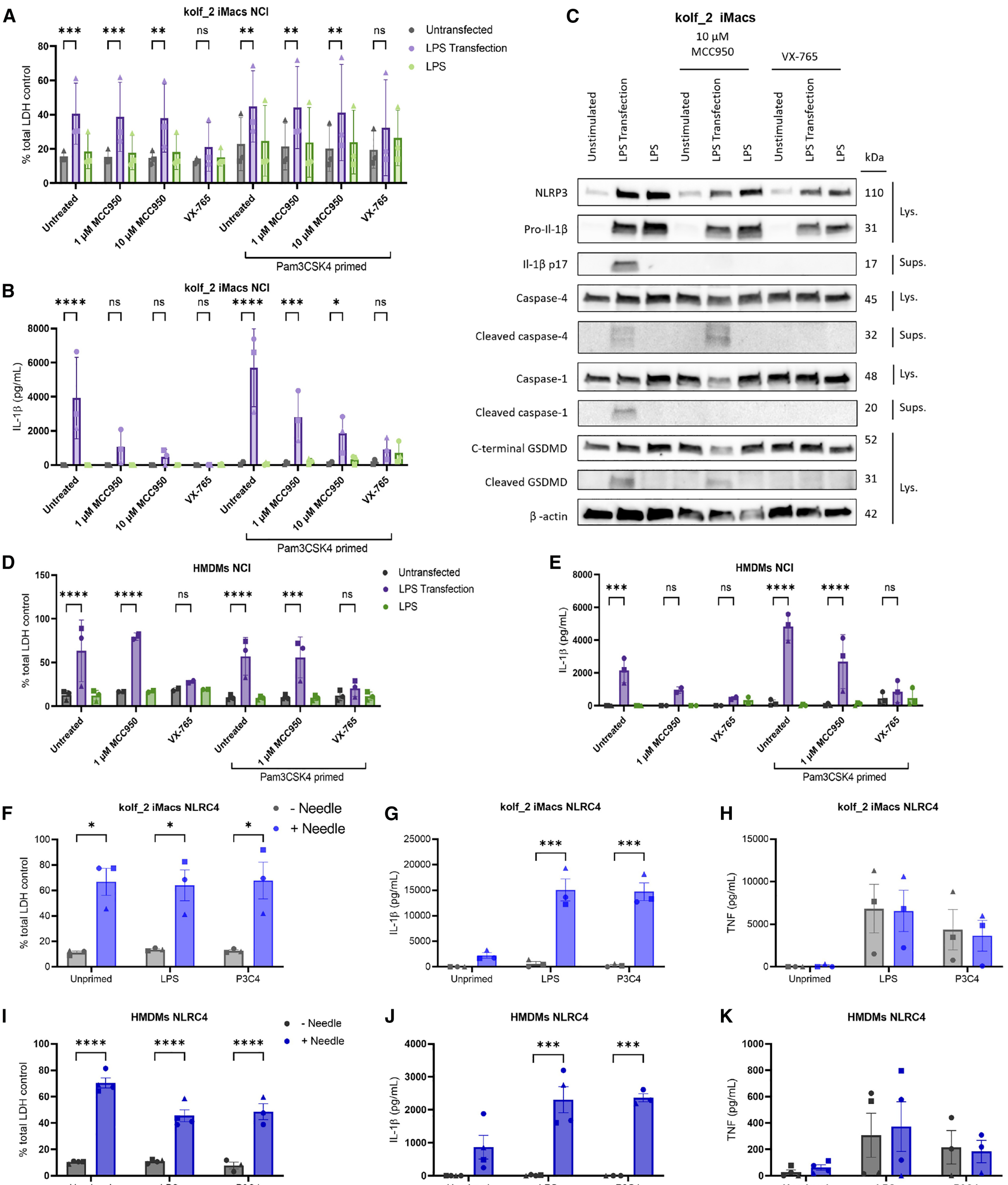

Figure 3. NCI and NLRC4 inflammasome activation is comparable in kolf_2 iMacs and HMDMs

(A–C) (A) LDH and (B) IL-1β release from kolf_2 iMacs in unprimed/Pam3CSK4-primed cells (16 h), and (C) western blot from unprimed kolf_2 iMacs following 5-h LPS transfection or stimulation ± MCC950/VX-765 30 min prior to transfection.

(legend continued on next page)

pyroptosis and significant IL-1β release, although IL-1β release is lower than in iMacs (Figures 3I–3K). Therefore, NLRC4 activation is similar between iMacs and HMDMs; however, iMacs release significantly higher levels of IL-1β.

Response to NLRP1/CARD8 stimulation

The function-to-find domain (FIIND) of NLRP1 and CARD8 inflammasomes must undergo proteolysis to allow inflammasome activation.[41] This can be triggered using the serine proteases dipeptidylpeptidase 8/9 (DPP8/9) inhibitor, talabostat (ValboroPro).[42] To study NLRP1/CARD8 responses in kolf_2 iMacs, cells were primed and treated with talabostat. Similar to previous work in T cells,[42] talabostat alone induces significant pyroptosis (Figure 4A). There is no significant pyroptosis in primed cells treated with talabostat, but there is significant IL-1β release (Figures 4A and 4B). Priming also triggers significant TNF release (Figure 4C). This contrasts with results from HMDMs where talabostat does not induce LDH or IL-1β release (Figures 4D and 4E). This is consistent with previous findings[43] and confirms that HMDMs are unresponsive to talabostat. The TNF response to priming is also reduced in HMDMs compared to iMacs (Figure 4F). In ieki_2 iMacs, anisomycin, which triggers NLRP1 activation through the ribotoxic stress response,[44] induces significant pyroptosis and IL-1β and TNF release (Figures 4G–4I). Interestingly, HMDMs also respond to anisomycin, with significant pyroptosis and IL-1β release (Figures 4J and 4K) and a further increase in TNF release in LPS-primed cells (Figure 4L).

NLRP1 interacts with ASC to activate caspase-1, while CARD8 interacts directly with caspase-1[45]; consequently, ASC specks are only present with NLRP1 activation. ASC specks are observed in all cells treated with talabostat, particularly in unprimed and Pam3CSK4-primed conditions (Figures 4M and 4N). In ieki_2 iMacs, anisomycin triggers significant ASC speck formation with/without priming (Figures S4B and S4C). Therefore, in iMacs, stimulation with talabostat or anisomycin activates NLRP1.

DISCUSSION

While some previous studies had observed inflammasome activation in iMacs, our aim was to comprehensively characterize a range of inflammasome responses in iMacs differentiated from different iPSC lines and benchmark them with HMDMs. In terms of macrophage marker expression and phagocytosis, iMacs generated here were very comparable to HMDMs. Previous work has characterized transcriptional responses to LPS in iMacs,[17] but as our interest is in protein complexes, we used a proteomics approach. We found that iMacs express high levels of inflammasome sensors and other inflammatory proteins and can thus form multiple inflammasomes. Our iMac proteomics dataset will be of interest for others interested in examining innate immune pathways using these cells.

NLRP3 was efficiently activated in iMacs; however, ATP induced a significantly lower response compared to nigericin in kolf_2 iMacs and HMDMs. This could be influenced by the polymorphic nature of the ATP receptor, P2X7R,[37] and comparison of the P2X7R gene in kolf_2 and ieki_2 cells supports this. In addition, sterile stimuli like ATP induce lower NLRP3 responses relative to microbial stimuli, reflecting the requirement for faster and stronger responses to limit pathogen dissemination.[34] Indeed, we observed that iMacs activate NLRP3 in response to nigericin alone. We do not detect IL-18 release in response to nigericin alone in kolf_2 iMacs; however, in ieki_2 iMacs, there is IL-18 release with nigericin alone at a similar level as the LPS-primed cells, indicating sex-specific differences in IL-18 release. Priming-independent NLRP3 activation by nigericin with cell death and IL-18 release has been reported in human monocytes, but not in macrophages.[46] HMDMs do not respond to nigericin alone, so this is a distinct response, potentially reflecting their more tissue resident-like ontogeny. iMac differentiation is independent of the transcription factor Myb, making iMacs developmentally more similar to tissue-resident macrophages than HMDMs.[47] In agreement, our proteomics dataset shows increased expression of some tissue-resident macrophage markers relative to HMDMs. Further work is needed to confirm whether the lower threshold for NLRP3 activation in iMacs is consistent with primary tissue-resident macrophages.

iMacs demonstrate robust responses to NCI and NAIP/NLRC4 activation similar to HMDMs. This includes high levels of pyroptosis in primed and unprimed cells in response to LPS transfection or Lfn-BsaL stimulation, confirming priming is not necessary for NCI or NAIP/NLRC4 activation. In human monocytes, caspase-4 is constitutively expressed[48]; however, studies have reported a requirement for IFN-γ priming to induce expression of granulocyte-binding proteins (GBPs) that are necessary for caspase-4 activation.[49] In iMacs, caspase-4 and GBP1–4 are constitutively expressed, which appears sufficient for NCI activation.

We observed differences in iMac NLRP1/CARD8 responses relative to HMDMs. In kolf_2 iMacs, talabostat causes NLRP1 activation, while HMDMs are unresponsive as previously reported.[43] Interestingly, ieki_2 iMacs and HMDMs respond to anisomycin, suggesting that NLRP1 inflammasome responses to ribotoxic stress are more sensitive than DPP8/9 inhibition. Human NLRP1 is predominantly found in keratinocytes and epithelial cells, while CARD8 is more common in leukocytes.[8,43] Proteomics analysis identified expression of NLRP1 and CARD8 in our iMacs; therefore, iMacs may be an attractive model for studying NLRP1 and CARD8. Future experiments can further explore this using NLRP1- and CARD8-specific stimuli.[42,50]

The "two-step" dogma of inflammasome activation was developed using mouse macrophages. Our results demonstrate there is no requirement for priming of NLRP3, NAIP/NLRC4, NLRP1, or NCI in human iMacs. Priming simply

(D and E) (D) LDH and (E) IL-1β release from HMDMs in unprimed/Pam3CSK4-primed cells (16 h) following 5-h LPS transfection or stimulation ± MCC950/VX-765 prior to transfection.

(F–K) LDH, IL-1β, and TNF release from (F–H) kolf_2 iMacs and (I–K) HMDMs following 4-h LPS/Pam3CSK4 and 2-h Lfn-BsaL plus PA.

Data represented as mean ± SEM, $n = 3$ kolf_2 replicates or $n = 3$–4 HMDM donors, *$p < 0.05$, **$p < 0.01$, ***$p < 0.001$, and ****$p < 0.0001$ by repeated measures ANOVA with Tukey's multiple comparisons (NCI) and two-way ANOVA with Šídák's multiple comparisons (NAIP/NLRC4).

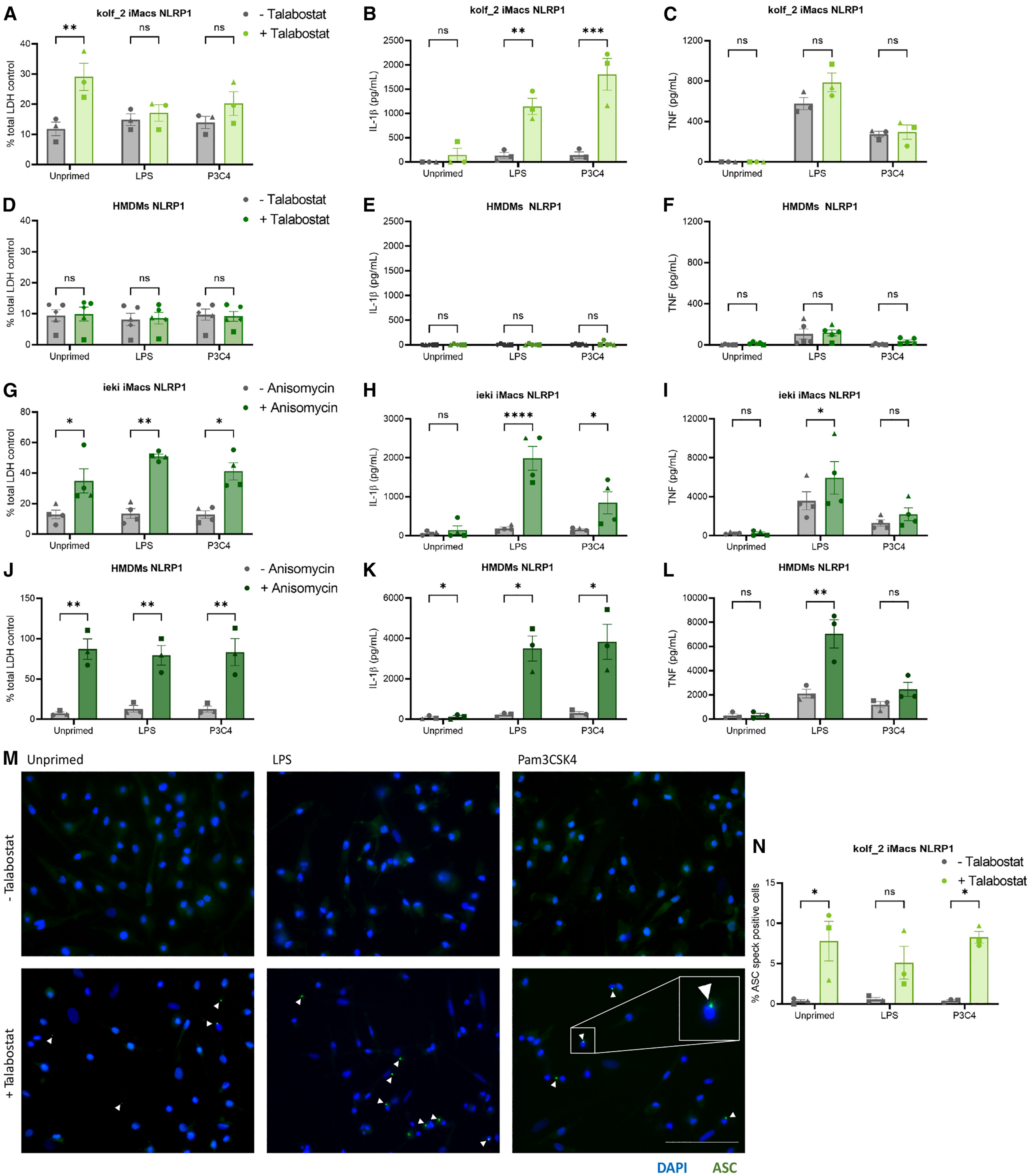

Figure 4. iMacs activate NLRP1 in response to talabostat, whereas both HMDMs and iMacs respond to anisomycin

(A–L) LDH, IL-1β, and TNF release from (A–C) kolf_2 iMacs and (D–F) HMDMs following 4-h LPS/Pam3CSK4 and 24-h talabostat and (G–I) ieki_2 iMacs and (J–L) HMDMs following 4-h LPS/Pam3CSK4 and 24-h anisomycin.

(M) Images from kolf_2 iMacs following 4-h LPS/Pam3CSK4 and 24-h talabostat. Scale bar, 100 μM.

(N) Quantification of DAPI+/speck+ cells.

Data represented as mean ± SEM, $n = 3/4$ kolf_2/ieki_2 replicates and $n = 3–5$ HMDM donors $^{*}p < 0.05$, $^{**}p < 0.01$, $^{***}p < 0.001$, $^{****}p < 0.0001$ by two-way ANOVA with Šídák's multiple comparisons.

increases pro-IL-1β, which is necessary for measuring IL-1β release. This increased sensitivity to stimuli likely reflects the necessity to provide rapid and robust anti-microbial responses in tissues.

The two iPSC lines, kolf_2 and ieki_2, are broadly comparable as both express high levels of macrophage markers, respond to TLR2 and TLR4 stimulation, and demonstrate functional inflammasome activation. Therefore, our results are widely applicable to studying inflammasome activity in macrophages derived from multiple iPSC lines.

In summary, we have shown that iMacs are an excellent model to study multiple inflammasomes. Unlike HMDMs, iMacs are genetically modifiable, providing a wide range of opportunities to study macrophage inflammasome function. iMacs may also be useful for studying tissue-resident macrophages, and protocols have been developed to differentiate specialized macrophages.[51] Recently, iMacs have been shown to reflect properties of disease-associated tissue-resident macrophages, including inflammatory bowel disease.[24] Future work in terminally differentiated macrophages could provide additional insight into the role of inflammasomes in specific tissues and disease states.

Limitations of the study

We performed key experiments using macrophages from male and female iPSC lines showing inflammasome responses are broadly conserved. However, more cell lines should be tested as genetic background could influence the results. We also focused on testing four well-characterized and therapeutically relevant inflammasomes but did not examine others such as pyrin. We did not study inflammasome responses in iMacs differentiated from alternative differentiation protocols; therefore, we cannot conclude that iMacs generated with all protocols will respond similarly. However, recent work comparing HMDMs to iMacs generated using an EB-independent protocol demonstrated similar expression of macrophage markers and response to LPS stimulation,[24] consistent with our findings. Therefore, we expect iMacs generally will respond to inflammasome stimuli in a similar manner as the iMacs generated here.

RESOURCE AVAILABILITY

Lead contact

Requests for additional information or mentioned resources should be sent to the lead contact, Rebecca Coll (r.coll@qub.ac.uk).

Materials availability

Any material and information used for this study are available from the lead contact upon request.

Data and code availability

- The iMac proteomics dataset was deposited on the PRIDE website (https://www.ebi.ac.uk/pride/login) and can be accessed using project accession PRIDE: PXD068076 and token: ilXveQUXDATv.
- This study does not report original code.
- Any additional information required to reanalyze the data reported in this paper is available from the lead contact upon request.

ACKNOWLEDGMENTS

This work was supported by funding from the Academy of Medical Sciences (SBR005\1104) (to R.C.C.), Biotechnology and Biological Sciences Research Council (BB/V016741/1) (to R.C.C. and B.C.C.), and Northern Ireland, Department for the Economy PhD studentships (to C.M.M., T.J.M., and E.C.M.). We would like to thank Dr. Subhankar Mukhopadhyay (King's College, London) for helpful discussions about iPSC-derived macrophage culture.

AUTHOR CONTRIBUTIONS

Conceptualization, R.C.C. and C.M.M.; funding acquisition, R.C.C. and B.C.C.; investigation, C.M.M., M.C., E.C.M., M.A., and T.J.M.; formal analysis, C.M.M., E.C.M., M.A., and B.C.C.; project administration, R.C.C.; supervision, R.C.C. and B.C.C.; visualization, C.M.M. and E.C.M.; writing – original draft, R.C.C. and C.M.M.; writing – review & editing, C.M.M., M.C., E.C.M., M.A., T.J.M., B.C.C., and R.C.C.

DECLARATION OF INTERESTS

R.C.C. is a co-inventor on patents and patent applications for NLRP3 inhibitors licensed to Inflazome Ltd., Ireland. R.C.C. is a consultant for BioAge Labs, USA (since 2020), and serves on the scientific advisory board of Viva In Vitro Diagnostics, Spain (since 2024). B.C.C. receives or has received research funding from Bruker Daltonics and Almac Discovery.

STAR★METHODS

Detailed methods are provided in the online version of this paper and include the following:

- KEY RESOURCES TABLE
- EXPERIMENTAL MODEL AND STUDY PARTICIPANT DETAILS
  - Culture and differentiation of induced pluripotent stem cell-derived macrophages
  - Isolation of CD14⁺ monocyte and differentiation of monocyte-derived macrophages
- METHOD DETAILS
  - Inflammasome activation
  - Flow cytometry
  - Phagocytosis assays
  - Mass spectrometry
  - Lactate dehydrogenase (LDH) assay
  - Enzyme-linked immunosorbent assay (ELISA)
  - Western blot
  - ASC speck staining and imaging
  - Polymorphism detection in RNA sequencing databases
- QUANTIFICATION AND STATISTICAL ANALYSIS

SUPPLEMENTAL INFORMATION

Supplemental information can be found online at https://doi.org/10.1016/j.crmeth.2026.101506.

Received: September 5, 2025
Revised: March 31, 2026
Accepted: May 27, 2026
Published: June 25, 2026

REFERENCES

1. Lamkanfi, M., and Dixit, V.M. (2014). Mechanisms and functions of inflammasomes. Cell *157*, 1013–1022. https://doi.org/10.1016/j.cell.2014.04.007.
2. McKee, C.M., and Coll, R.C. (2020). NLRP3 inflammasome priming: A riddle wrapped in a mystery inside an enigma. J. Leukoc. Biol. *108*, 937–952. https://doi.org/10.1002/JLB.3MR0720-513R.
3. Li, H., Guan, Y., Liang, B., Ding, P., Hou, X., Wei, W., and Ma, Y. (2022). Therapeutic potential of MCC950, a specific inhibitor of NLRP3

inflammasome. Eur. J. Pharmacol. *928*, 175091. https://doi.org/10.1016/j.ejphar.2022.175091.

4. Mullard, A. (2019). NLRP3 inhibitors stoke anti-inflammatory ambitions. Nat. Rev. Drug Discov. *18*, 405–407. https://doi.org/10.1038/d41573-019-00086-9.
5. Awad, F., Assrawi, E., Jumeau, C., Georgin-Lavialle, S., Cobret, L., Duquesnoy, P., Piterboth, W., Thomas, L., Stankovic-Stojanovic, K., Louvrier, C., et al. (2017). Impact of human monocyte and macrophage polarization on NLR expression and NLRP3 inflammasome activation. PLoS One *12*, e0175336. https://doi.org/10.1371/journal.pone.0175336.
6. Mestas, J., and Hughes, C.C.W. (2004). IMMUNOLOGY Of Mice and Not Men: Differences between Mouse and Human Immunology. J. Immunol. *172*, 2731–2738. https://doi.org/10.4049/jimmunol.172.5.2731.
7. Lilue, J., Doran, A.G., Fiddes, I.T., Abrudan, M., Armstrong, J., Bennett, R., Chow, W., Collins, J., Collins, S., Czechanski, A., et al. (2018). Sixteen diverse laboratory mouse reference genomes define strain-specific haplotypes and novel functional loci. Nat. Genet. *50*, 1574–1583. https://doi.org/10.1038/s41588-018-0223-8.
8. Johnson, D.C., Taabazuing, C.Y., Okondo, M.C., Chui, A.J., Rao, S.D., Brown, F.C., Reed, C., Peguero, E., de Stanchina, E., Kentsis, A., and Bachovchin, D.A. (2018). DPP8/DPP9 inhibitor-induced pyroptosis for treatment of acute myeloid leukemia. Nat. Med. *24*, 1151–1156. https://doi.org/10.1038/s41591-018-0082-y.
9. Egan, M.S., Zhang, J., and Shin, S. (2023). Human and mouse NAIP/NLRC4 inflammasome responses to bacterial infection. Curr. Opin. Microbiol. *73*, 102298. https://doi.org/10.1016/J.MIB.2023.102298.
10. Indramohan, M., Stehlik, C., and Dorfleutner, A. (2018). COPs and POPs Patrol Inflammasome Activation. J. Mol. Biol. *430*, 153–173. https://doi.org/10.1016/J.JMB.2017.10.004.
11. Wilhelmsen, K., Deshpande, A., Tronnes, S., Mahanta, M., Banicki, M., Cochran, M., Cowdin, S., Fortney, K., Hartman, G., Hughes, R.E., et al. (2025). Discovery of potent and selective inhibitors of human NLRP3 with a novel mechanism of action. J. Exp. Med. *222*, e20242403. https://doi.org/10.1084/JEM.20242403.
12. Chanput, W., Mes, J.J., and Wichers, H.J. (2014). THP-1 cell line: An in vitro cell model for immune modulation approach. Int. Immunopharmacol. *23*, 37–45. https://doi.org/10.1016/J.INTIMP.2014.08.002.
13. Giambelluca, S., Ochs, M., and Lopez-Rodriguez, E. (2022). Resting time after phorbol 12-myristate 13-acetate in THP-1 derived macrophages provides a non-biased model for the study of NLRP3 inflammasome. Front. Immunol. *13*, 958098. https://doi.org/10.3389/fimmu.2022.958098.
14. Tedesco, S., De Majo, F., Kim, J., Trenti, A., Trevisi, L., Fadini, G.P., Bolego, C., Zandstra, P.W., Cignarella, A., and Vitiello, L. (2018). Convenience versus biological significance: Are PMA-differentiated THP-1 cells a reliable substitute for blood-derived macrophages when studying in vitro polarization? Front. Pharmacol. *9*, 71. https://doi.org/10.3389/FPHAR.2018.00071/BIBTEX.
15. Adati, N., Huang, M.C., Suzuki, T., Suzuki, H., and Kojima, T. (2009). High-resolution analysis of aberrant regions in autosomal chromosomes in human leukemia THP-1 cell line. BMC Res. Notes *2*, 153. https://doi.org/10.1186/1756-0500-2-153.
16. Mukherjee, C., Hale, C., and Mukhopadhyay, S. (2018). A simple multistep protocol for differentiating human induced pluripotent stem cells into functional macrophages. Methods Mol. Biol. *1784*, 13–28. https://doi.org/10.1007/978-1-4939-7837-3_2.
17. Alasoo, K., Martinez, F.O., Hale, C., Gordon, S., Powrie, F., Dougan, G., Mukhopadhyay, S., and Gaffney, D.J. (2015). Transcriptional profiling of macrophages derived from monocytes and iPS cells identifies a conserved response to LPS and novel alternative transcription. Sci. Rep. *5*, 12524. https://doi.org/10.1038/srep12524.
18. Hale, C., Yeung, A., Goulding, D., Pickard, D., Alasoo, K., Powrie, F., Dougan, G., and Mukhopadhyay, S. (2015). Induced pluripotent stem cell derived macrophages as a cellular system to study Salmonella and other pathogens. PLoS One *10*, e0124307. https://doi.org/10.1371/journal.pone.0124307.
19. Choi, K.D., Vodyanik, M., and Slukvin, I.I. (2011). Hematopoietic differentiation and production of mature myeloid cells from human pluripotent stem cells. Nat. Protoc. *6*, 296–313. https://doi.org/10.1038/nprot.2010.184.
20. Senju, S., Haruta, M., Matsumura, K., Matsunaga, Y., Fukushima, S., Ikeda, T., Takamatsu, K., Irie, A., and Nishimura, Y. (2011). Generation of dendritic cells and macrophages from human induced pluripotent stem cells aiming at cell therapy. Gene Ther. *18*, 874–883. https://doi.org/10.1038/gt.2011.22.
21. Kambal, A., Mitchell, G., Cary, W., Gruenloh, W., Jung, Y., Kalomoiris, S., Nacey, C., McGee, J., Lindsey, M., Fury, B., et al. (2011). Generation of HIV-1 resistant and functional macrophages from hematopoietic stem cell-derived induced pluripotent stem cells. Mol. Ther. *19*, 584–593. https://doi.org/10.1038/mt.2010.269.
22. van Wilgenburg, B., Browne, C., Vowles, J., and Cowley, S.A. (2013). Efficient, Long Term Production of Monocyte-Derived Macrophages from Human Pluripotent Stem Cells under Partly-Defined and Fully-Defined Conditions. PLoS One *8*, e71098. https://doi.org/10.1371/JOURNAL.PONE.0071098.
23. Douthwaite, H., Arteagabeitia, A.B., and Mukhopadhyay, S. (2022). Differentiation of Human Induced Pluripotent Stem Cell into Macrophages. Bio. Protoc. *12*, e4361. https://doi.org/10.21769/BioProtoc.4361.
24. Sauer, J., Schloesser, D., Koeppen, K., Fundel-Clemens, K., Simone-Roach, C., Hall, K., Ivovic, A., Makings, J., Reiss, D., Traub, M., et al. (2025). iPSC-derived macrophages: An in vitro model to study human disease-relevant macrophage biology. J. Immunol. *214*, 3115–3130. https://doi.org/10.1093/JIMMUN/VKAF184.
25. Nenasheva, T., Gerasimova, T., Serdyuk, Y., Grigor'eva, E., Kosmiadi, G., Nikolaev, A., Dashinimaev, E., and Lyadova, I. (2020). Macrophages Derived From Human Induced Pluripotent Stem Cells Are Low-Activated "Naïve-Like" Cells Capable of Restricting Mycobacteria Growth. Front. Immunol. *11*, 1016. https://doi.org/10.3389/FIMMU.2020.01016/BIBTEX.
26. Zhang, H., and Reilly, M.P. (2017). Human induced pluripotent stem cell-derived macrophages for unraveling human macrophage biology. Arterioscler. Thromb. Vasc. Biol. *37*, 2000–2006. https://doi.org/10.1161/ATVBAHA.117.309195.
27. Chow, S.H., Deo, P., Yeung, A.T.Y., Kostoulias, X.P., Jeon, Y., Gao, M.L., Seidi, A., Olivier, F.A.B., Sridhar, S., Nethercott, C., et al. (2020). Targeting NLRP3 and staphylococcal pore-forming toxin receptors in human-induced pluripotent stem cell-derived macrophages. J. Leukoc. Biol. *108*, 967–981. https://doi.org/10.1002/JLB.4MA0420-497R.
28. Seki, R., Ohta, A., Niwa, A., Sugimine, Y., Naito, H., Nakahata, T., and Saito, M.K. (2020). Induced pluripotent stem cell-derived monocytic cell lines from a NOMID patient serve as a screening platform for modulating NLRP3 inflammasome activity. PLoS One *15*, e0237030. https://doi.org/10.1371/journal.pone.0237030.
29. Schmacke, N.A., O'Duill, F., Gaidt, M.M., Szymanska, I., Kamper, J.M., Schmid-Burgk, J.L., Mädler, S.C., Mackens-Kiani, T., Kozaki, T., Chauhan, D., et al. (2022). IKKβ primes inflammasome formation by recruiting NLRP3 to the trans-Golgi network. Immunity *55*, 2271–2284.e7. https://doi.org/10.1016/J.IMMUNI.2022.10.021.
30. Alasoo, K., Rodrigues, J., Mukhopadhyay, S., Knights, A.J., Mann, A.L., Kundu, K., Hale, C., Dougan, G., and Gaffney, D.J. (2018). Shared genetic effects on chromatin and gene expression indicate a role for enhancer priming in immune response. Nat Gen *50*, 424–431. https://doi.org/10.1038/S41588-018-0046-7.
31. Monkley, S., Krishnaswamy, J.K., Göransson, M., Clausen, M., Meuller, J., Thörn, K., Hicks, R., Delaney, S., and Stjernborg, L. (2020). Optimised generation of iPSC-derived macrophages and dendritic cells that are functionally and transcriptionally similar to their primary counterparts. Zambidis ET. PLoS One *15*, e0243807. https://doi.org/10.1371/journal.pone.0243807.

32. Hirayama, D., Iida, T., and Nakase, H. (2017). The Phagocytic Function of Macrophage-Enforcing Innate Immunity and Tissue Homeostasis. Int. J. Mol. Sci. *19*, 92. https://doi.org/10.3390/IJMS19010092.

33. Planès, R., Pinilla, M., Santoni, K., Hessel, A., Passemar, C., Lay, K., Paillette, P., Valadão, A.C., Robinson, K.S., Bastard, P., et al. (2022). Human NLRP1 is a sensor of pathogenic coronavirus 3CL proteases in lung epithelial cells. Mol. Cell *82*, 1–16. https://doi.org/10.1016/J.MOLCEL.2022.04.033.

34. Bezbradica, J.S., Coll, R.C., and Schroder, K. (2017). Sterile signals generate weaker and delayed macrophage NLRP3 inflammasome responses relative to microbial signals. Cell. Mol. Immunol. *14*, 118–126. https://doi.org/10.1038/cmi.2016.11.

35. Dick, S.A., Wong, A., Hamidzada, H., Nejat, S., Nechanitzky, R., Vohra, S., Mueller, B., Zaman, R., Kantores, C., Aronoff, L., et al. (2022). Three tissue resident macrophage subsets coexist across organs with conserved origins and life cycles. Sci. Immunol. *7*, 7777. https://doi.org/10.1126/sciimmunol.abf7777.

36. Swanson, K.V., Deng, M., and Ting, J.P.Y. (2019). The NLRP3 inflammasome: molecular activation and regulation to therapeutics. Nat. Rev. Immunol. *19*, 477–489. https://doi.org/10.1038/s41577-019-0165-0.

37. Semple, C., Choi, K.Y.G., Kroeker, A., Denechezhe, L., Orr, P., Mookherjee, N., and Larcombe, L. (2019). Polymorphisms in the P2X7 receptor, and differential expression of Toll-like receptor-mediated cytokines and defensins, in a Canadian Indigenous group. Sci. Rep. *9*, 14204. https://doi.org/10.1038/s41598-019-50596-0.

38. Shi, X., Sun, Q., Hou, Y., Zeng, H., Cao, Y., Dong, M., Ding, J., and Shao, F. (2023). Recognition and maturation of IL-18 by caspase-4 noncanonical inflammasome. Nature *624*, 442–450. https://doi.org/10.1038/s41586-023-06742-w.

39. Ross, C., Chan, A.H., Von Pein, J., Boucher, D., and Schroder, K. (2018). Dimerization and auto-processing induce caspase-11 protease activation within the non-canonical inflammasome. Life Sci. Alliance *1*, e201800237. https://doi.org/10.26508/lsa.201800237.

40. Hu, Z., Zhou, Q., Zhang, C., Fan, S., Cheng, W., Zhao, Y., Shao, F., Wang, H.W., Sui, S.F., and Chai, J. (2015). Structural and biochemical basis for induced self-propagation of NLRC4. Science *350*, 399–404. https://doi.org/10.1126/science.aac5489.

41. Sandstrom, A., Mitchell, P.S., Goers, L., Mu, E.W., Lesser, C.F., and Vance, R.E. (2019). Functional degradation: A mechanism of NLRP1 inflammasome activation by diverse pathogen enzymes. Science *364*, eaau1330. https://doi.org/10.1126/science.aau1330.

42. Bauernfried, S., Scherr, M.J., Pichlmair, A., Duderstadt, K.E., and Hornung, V. (2021). Human NLRP1 is a sensor for double-stranded RNA. Science *371*, eabd0811. https://doi.org/10.1126/science.abd0811.

43. Linder, A., Bauernfried, S., Cheng, Y., Albanese, M., Jung, C., Keppler, O.T., and Hornung, V. (2020). CARD8 inflammasome activation triggers pyroptosis in human T cells. EMBO J. *39*, e105071. https://doi.org/10.15252/embj.2020105071.

44. Jenster, L.M., Lange, K.E., Normann, S., vom Hemdt, A., Wuerth, J.D., Schiffelers, L.D.J., Tesfamariam, Y.M., Gohr, F.N., Klein, L., Kaltheuner, I.H., et al. (2022). P38 kinases mediate NLRP1 inflammasome activation after ribotoxic stress response and virus infection. J. Exp. Med. *220*, e20220837. https://doi.org/10.1084/jem.20220837.

45. Gong, Q., Robinson, K., Xu, C., Huynh, P.T., Chong, K.H.C., Tan, E.Y.J., Zhang, J., Boo, Z.Z., Teo, D.E.T., Lay, K., et al. (2021). Structural basis for distinct inflammasome complex assembly by human NLRP1 and CARD8. Nat. Commun. *12*, 188. https://doi.org/10.1038/s41467-020-20319-5.

46. Gritsenko, A., Yu, S., Martin-Sanchez, F., Diaz-del-Olmo, I., Nichols, E.M., Davis, D.M., Brough, D., and Lopez-Castejon, G. (2020). Priming Is Dispensable for NLRP3 Inflammasome Activation in Human Monocytes In Vitro. Front. Immunol. *11*, 565924. https://doi.org/10.3389/fimmu.2020.565924.

47. Buchrieser, J., James, W., and Moore, M.D. (2017). Human Induced Pluripotent Stem Cell-Derived Macrophages Share Ontogeny with MYB-Independent Tissue-Resident Macrophages. Stem Cell Rep. *8*, 334–345. https://doi.org/10.1016/J.STEMCR.2016.12.020.

48. Yang, J., Zhao, Y., and Shao, F. (2015). Non-canonical activation of inflammatory caspases by cytosolic LPS in innate immunity. Curr. Opin. Immunol. *32*, 78–83. https://doi.org/10.1016/j.coi.2015.01.007.

49. Santos, J.C., Boucher, D., Schneider, L.K., Demarco, B., Dilucca, M., Shkarina, K., Heilig, R., Chen, K.W., Lim, R.Y.H., and Broz, P. (2020). Human GBP1 binds LPS to initiate assembly of a caspase-4 activating platform on cytosolic bacteria. Nat. Commun. *11*, 3276. https://doi.org/10.1038/s41467-020-16889-z.

50. Chen, Q., Wang, A., Covelli, D.J., Bhattacharjee, A., Wang, Q., Orth-He, E.L., Rao, S.D., Huang, H.C., Ball, D.P., Hsiao, J.C., and Bachovchin, D.A. (2023). Optimized M24B Aminopeptidase Inhibitors for CARD8 Inflammasome Activation. J. Med. Chem. *66*, 2589–2607. https://doi.org/10.1021/acs.jmedchem.2c01535.

51. Washer, S.J., Perez-Alcantara, M., Chen, Y., Steer, J., James, W.S., Trynka, G., Bassett, A.R., and Cowley, S.A. (2022). Single-cell transcriptomics defines an improved, validated monoculture protocol for differentiation of human iPSC to microglia. Sci. Rep. *12*, 19454. https://doi.org/10.1038/s41598-022-23477-2.

52. Kverneland, A.H., Harking, F., Vej-Nielsen, J.M., Huusfeldt, M., Bekker-Jensen, D.B., Svane, I.M., Bache, N., and Olsen, J.V. (2024). Fully Automated Workflow for Integrated Sample Digestion and Evotip Loading Enabling High-Throughput Clinical Proteomics. Mol. Cell. Proteomics *23*, 100790. https://doi.org/10.1016/J.MCPRO.2024.100790.

53. Meier, F., Brunner, A.D., Frank, M., Ha, A., Bludau, I., Voytik, E., Kaspar-Schoenefeld, S., Lubeck, M., Raether, O., Bache, N., et al. (2020). diaPASEF: parallel accumulation–serial fragmentation combined with data-independent acquisition. Nat. Methods *17*, 1229–1236. https://doi.org/10.1038/s41592-020-00998-0.

54. Skowronek, P., Thielert, M., Voytik, E., Tanzer, M.C., Hansen, F.M., Willems, S., Karayel, O., Brunner, A.D., Meier, F., and Mann, M. (2022). Rapid and In-Depth Coverage of the (Phospho-)Proteome With Deep Libraries and Optimal Window Design for dia-PASEF. Mol. Cell. Proteomics *21*, 100279. https://doi.org/10.1016/J.MCPRO.2022.100279.

55. Penny, J., Arefian, M., Schroeder, G.N., Bengoechea, J.A., and Collins, B.C. (2023). A gas phase fractionation acquisition scheme integrating ion mobility for rapid diaPASEF library generation. Proteomics *23*, 2200038. https://doi.org/10.1002/pmic.202200038.

56. Kulak, N.A., Pichler, G., Paron, I., Nagaraj, N., and Mann, M. (2014). Minimal, encapsulated proteomic-sample processing applied to copy-number estimation in eukaryotic cells. Nat. Methods *11*, 319–324. https://doi.org/10.1038/nmeth.2834.

STAR★METHODS

KEY RESOURCES TABLE

REAGENT or RESOURCE	SOURCE	IDENTIFIER
Antibodies		
Anti-human CD14, FITC	BioRad	MCA1568A488; RRID:AB_3100368
IgG2a Negative control, FITC	BioRad	MCA929A488; RRID:AB_324436
Anti-human CD16, PE/Cyanine7	Biolegend	302016; RRID:AB_314216
IgG1 kappa Isotype Control, PE/Cyanine7	Biolegend	1400126; RRID:AB_326448
Anti-human CD44, APC	BioRad	MCA89A647T; RRID:AB_1102041
IgG2a Negative Control, APC	BioRad	MCA929A647T; RRID:AB_322306
Anti-human CD64, PE	BioRad	MCA756PE; RRID:AB_321800
IgG1 Negative Control, PE	BioRad	MCA928PE; RRID:AB_322263
Anti-human CD68, PE	BioRad	MCA6014PE; RRID:AB_3101254
IgG2b Negative Control, PE	BioRad	MCA691PE; RRID:AB_322277
Fixable Viability Dye eFluor	eBioscience	65-0866-14
LysoTracker red DND-99	ThermoFisher	L7528
Anti-β-actin	Sigma-Aldrich	A2228; RRID:AB_476697)
Anti-caspase-1 (D7F10)	Cell Signaling Technology	3866; RRID:AB_2069051
Anti-cleaved caspase-1	Cell Signaling Technology	4199S; RRID:AB_1903916
Anti-caspase-4	Cell Signaling Technology	4450S; RRID:AB_1950386
Anti-GSDMD N-terminal	Abcam	ab215203; RRID:AB_2916166
Anti-GSDMD C-terminal	Abcam	ab210070; RRID:AB_2893325
Anti-IL-1β	R&D Systems	AF-401-NA; RRID:AB_416684
Anti-NLRP3 (Cryo-2)	Adipogen	AG-20B-0014; RRID:AB_2490202
Anti-NLRP3 (D4D8T)	Cell Signaling Technology	15101; RRID:AB_2722591
HRP- Anti-Rabbit IgG	Jackson Immunoresearch	111-035-003; RRID:AB_2313567
HRP- Anti-Goat IgG	Jackson Immunoresearch	705-035-147; RRID:AB_2313587
HRP- Anti-Mouse IgG	Jackson Immunoresearch	515-035-003; RRID:AB_2340295
Anti-ASC	BioLegend	676502; RRID:AB_2565643
Alexa Fluor™ 488 Donkey anti-mouse	Invitrogen	A21202; RRID:AB_141607
Chemicals, peptides, and recombinant proteins		
Vitronectin	ThermoFisher	A14700
StemFlex medium	ThermoFisher	A3349401
Rho-kinase (Rock) inhibitor Y- 27632 Dihydrochloride	Merck	Y0503
UltraPure™ EDTA, pH 8.0	ThermoFisher	15575020
DPBS (10×), no calcium, no magnesium	ThermoFisher	14200075
BMP-4	R&D Systems	314-BP010
Gelatine	Sigma-Aldrich	G1890
Water for embryo transfer	Sigma-Aldrich	W1503
X-VIVO 15 Serum Free Medium	Lonza	BE02-060F
GlutaMAX	ThermoFisher	35050061
Penicillin-streptomycin	ThermoFisher	15140122
M-CSF	Proteintech	HZ-1192
Lidocaine monohydrate	Sigma-Aldrich	L5647-100g
RMPI 1640	ThermoFisher	21870076
Fetal bovine serum (FBS)	ThermoFisher	26140079
Ultrapure LPS from E. coli K12	InvivoGen	tlrl-peklps

(*Continued on next page*)

Continued

REAGENT or RESOURCE	SOURCE	IDENTIFIER
Triacylated lipopeptide Pam3CSK4	InvivoGen	tlrl-pms
Nigericin sodium salt	AdipoGen	AGCN2-0020-M005
Adenosine 5′-Triphosphate (ATP) disodium salt	Merck Milipore	1191-1GM
Lipofectamine LTX Reagent with PLUS Reagent	ThermoFisher	A12621
Talabostat mesylate	R&D Systems	3719/10
Lfn-BsaL (a fusion protein of the N-terminal domain of the Bacillus anthracis lethal factor and BsaL needle protein from Burkholderia Pseudomallei)	InvivoGen	tlrl-ndl
Anthrax Protective Antigen (PA), Recombinant from Bacillus anthracis	Quadratech Diagnostics	LL-171E
Human TruStain FXX	Biolegend	422302
eBioscience™ Intracellular Fixation & Permeabilization Buffer Set	ThermoFisher	88-8824-00
OptiMEM	ThermoFisher	31985070
Latex amine-modified polystyrene, fluorescent yellow-green beads	Sigma-Aldrich	L1030-1ML
4% paraformaldehyde (PFA)	Merck Life Science	818715
Triton X-100	Sigma-Aldrich	T9284-500ML
Bovine serum albumin (BSA)	Sigma-Aldrich	A7906-100G
Vectashield Antifade Mounting Medium with DAPI	Vector Laboratories	H-1299
Phosphate-buffered saline (PBS)	Sigma-Aldrich	P4417-50TAB
Tween®20	Sigma-Aldrich	P7949-500ML
BaseMuncher	Expedeon	BM0025
NuPAGE LDS sample buffer	Invitrogen	NP0007
Dithiothreitol (DTT)	Merck Life Science	20–265
4–20% Mini-PROTEAN® TGX™ Precast Protein Gels, 10-well	BioRad	4561093
Tris	Sigma-Aldrich	T6066-1KG
Glycine	Melford	56406
Sodium dodecyl sulfate (SDS)	Merck	L3771
Precision Plus Protein Kaleidoscope Standard	BioRad	1610375EDU
Nitrocellulose membrane	BioRad	1620115
Filter paper	BioRad	1704085
Trans-Blot Turbo 5× Transfer Buffer	BioRad	10026938
Ethanol	Fisher Scientific	16685992
Sodium chloride	Fisher Scientific	S/3120/65
Fat-free powdered milk	Marvel	N/A
Clarity ECL	Biorad	170–5061
SuperSignal™ West Atto	Thermo Fisher	A38555
Hydrogen peroxide	Sigma-Aldrich	16911-1L-F
Sodium deoxycholate	Sigma-Aldrich	D6750-100G
Tris (2-carboxyethyl) phosphine hydrochloride (TCEP)	Sigma-Aldrich	C4706-2G
2-Chloroacetamide ≥98.0% (HPLC)	Sigma-Aldrich	22790-250G-F
MagReSyn Hydroxyl beads	ReSyn Biosciences	MR-HYX100
Acetonitrile (ACN)	Sigma-Aldrich	302031-100mL

(*Continued on next page*)

Continued

REAGENT or RESOURCE	SOURCE	IDENTIFIER
Triethylammonium bicarbonate buffer (TEAB)	Sigma-Aldrich	T7408
Lys-C	Thermo Fisher	90397
Trypsin	Pierce	90058
Critical commercial assays		
Cytotoxicity Detection Kit Roche	Sigma-Aldrich	11644793001
LDH-Glo Cytotoxicity Assay	Promega	J2381
Human IL-1 beta/IL-1F2 DuoSet ELISA	R&D Systems	DY201
Human TNF-alpha DuoSet ELISA	R&D Systems	DY210
Human IL-18 ELISA kit	ThermoFisher	BMS267-2MST
Pierce^{TM} Rapid Gold BCA Protein Assay Kit	ThermoFisher	A53226
Deposited data		
Proteomics dataset	PRIDE website	https://www.ebi.ac.uk/pride/login: Project accession PRIDE: PXD068076 Token: ilXveQUXDATv
Experimental models: Cell lines		
Human induced pluripotent stem cell line	Wellcome Sanger Institute	HPSI0114i-kolf_2-C1 and HPSI1113i-ieki_2
Software and algorithms		
Biorender	Biorender Software	https://biorender.com/
Excel	Microsoft Office	https://www.office.com/
Graphpad Prism 10	GraphPad Software	https://www.graphpad.com/
Affinity designer	Serif	https://affinity.serif.com
FlowJo	BD Biosciences	https://www.flowjo.com/
Leica LAS X	Leica LAS X software	https://www.leica-microsystems.com/products/microscope-software/p/leica-las-x-ls/
Fiji ImageJ	ImageJ Software	https://imagej.net/software/fiji/
Spectronaut	Biognosys	https://biognosys.com/software/spectronaut/
RStudio	RStudio	https://posit.co/download/rstudio-desktop/
Other		
10 cm non-treated cell culture dish	Sarstedt	83.3900
6 well plate	Sarstedt	83.3920
24 well plate	Sarstedt	83.3922
96 well plate	Sarstedt	83.3924
T175 flask	Sarstedt	83.3912.002
Low-adherent, U-bottom 96 well plate	Aquilant	650970
Uncoated ELISA plate	VWR International	735–0199
Eppendorf microcentrifuge tubes (1.5 mL)	Sarstedt	72.690.001
Centrifuge tubes (15 mL)	Sarstedt	62.554.502
Centrifuge tubes (50 mL)	Sarstedt	62.547.254
Pipette tips (10 µL)	Sarstedt	70.1130
Pipette tips (200 µL)	Sarstedt	70.3030
Pipette tips (1000 µL)	Sarstedt	70.3050
Serological Pipette 25 mL	Sarstedt	86.1685.001
Serological Pipette 10 mL	Sarstedt	86.1254.001
Serological Pipette 5 mL	Sarstedt	86.1253.001
Tube 50 mL	Sarstedt	62.547.254
Tube 15 mL	Sarstedt	62.554.502

(*Continued on next page*)

Continued

REAGENT or RESOURCE	SOURCE	IDENTIFIER
Cell Scraper	Sarstedt	83.3951
Coverslips	VWR International	631–0149
Evotips	Evosep	EV2011
Laminar flow tissue culture hood	Contained Air Solutions	N/A
Tissue culture incubator	Panasonic Healthcare Co	N/A
FACS Canto II cytometer	Becton Dickenson	N/A
Stellaris-5 confocal microscope	Leica Microsystems	N/A
FLUOStar Omega plate reader	BMG Biotech	https://www.bmglabtech.com/en/fluostar-omega/
Mini-PROTEAN Tetra Vertical Electrophoresis Cell	BioRad	1658004
Trans-Blot® Turbo™ Transfer System	BioRad	1704150EDU
G:BOX	SYNGENE	N/A
DM5500 fluorescent microscope	Leica Microsystems	N/A
TimsTOF HT quadrupole time-of-flight mass spectrometer	Bruker	N/A

EXPERIMENTAL MODEL AND STUDY PARTICIPANT DETAILS

Culture and differentiation of induced pluripotent stem cell-derived macrophages

Kolf_2 and ieki_2 iPSCs were obtained from the Wellcome Sanger Institute (Hinxton, UK). iPSCs were thawed and plated onto vitronectin (ThermoFisher, Cat # A14700) coated plates in StemFlex (ThermoFisher, Cat # A3349401) with 10 μM Rho-kinase (Rock) inhibitor Y- 27632 Dihydrochloride (Merck, Cat #Y0503). Cells were cultured in tissue culture incubators at 37°C with 5% CO2 in a humidified atmosphere. Medium was changed daily (without Rock inhibitor) until cells were 70–80% confluent. Cells were passaged using 0.5 mM PBS-EDTA and plated onto vitronectin-coated 10 cm2 tissue culture dishes in StemFlex medium with 10 μM Rock inhibitor.

To generate embryoid bodies (EBs), iPSCs were harvested and seeded onto a low-adherent, U-bottom 96 well plate at 0.5×10^5 per well in 100 μL in StemFlex medium supplemented with 50 ng/mL BMP-4 (R&D Systems, Cat # 314-BP010) and 10 μM Rock inhibitor. The 96 well plates were centrifuged for 3 min at 300 G and incubated for 5 days to form EBs.

The EBs were transferred to gelatine (Sigma-Aldrich, Cat #G1890) coated tissue culture flasks in myeloid precursor base medium (X-VIVO 15 Serum Free Medium; Lonza, Cat # BE02-060F, supplemented with 2 mM GlutaMAX; ThermoFisher, Cat # 35050061, 100 IU/mL penicillin-streptomycin; ThermoFisher, Cat # 15140122, 50 ng/mL M-CSF; Proteintech, Cat # HZ-1192 and 25 ng/mL IL-3; Peprotech, Cat # 200-03-250UG). Media was changed every 4–5 days. Approximately 3–4 weeks after transferring the EBs to the gelatine-coated plates, they began to produce myeloid precursor cells which were then harvested and plated in iMac medium (RMPI 1640; ThermoFisher, Cat # 21870076 supplemented with 1-% fetal bovine serum; ThermoFisher, Cat # 26140079, 2 mM GlutaMAX, 100 IU/mL penicillin-streptomycin, and 100 ng/mL M-CSF) and differentiated for 7 days. Differentiated iMacs were harvested using Lidocaine monohydrate (Sigma-Aldrich, Cat #L5647-100g) and plated onto 96-, 24-, and 6-well plates at 0.5×10^5 in 100 μL, 1×10^5 in 500 μL, and 1×10^6 cells in 1 mL per well respectively in iMac medium.

Isolation of CD14$^+$ monocyte and differentiation of monocyte-derived macrophages

Buffy coats were obtained from the Northern Ireland Blood Transfusion Service (NIBTS reference number 2019/05), ethical approval for the use of buffy coats was from the Queen's University Faculty Research Ethics Committee (reference MHLS 19_17). Basic characteristics of buffy coat donors used are outlined in Table S2. Peripheral blood mononuclear cells (PBMCs) were isolated from human buffy coats by Ficoll–Paque PLUS (Sigma Aldrich, Cat # 17-1440-03) density centrifugation for 30 min at 200 G, room temperature, with the brake set to slow. PBMCs were incubated with CD14 magnetic microbeads (Miltenyi Biotech, Cat # 130-118-906) at 4°C with rotation and CD14$^+$ monocytes isolated by positive magnetic selection according to manufacturer's instructions (Miltenyi Biotec). CD14$^+$ monocytes were differentiated into macrophages with 50 ng/mL M-CSF for 7 days in HMDM medium (RPMI 1640 medium containing 10% FBS, 100 IU/mL penicillin/streptomycin, 1 × GlutaMAX, and 1 mM sodium pyruvate; Life Technologies, Cat # 11360070) at 37°C in a humidified 5% CO2 incubator. HMDM were harvested by scraping and seeded onto 96-, 24-, and 6-well plates at 0.7×10^5 in 100 μL, 1×10^5 in 500 μL, and 1×10^6 in 1 mL per well in HMDM medium.

METHOD DETAILS

Inflammasome activation

To activate NLRP3, cells were primed with 100 ng/mL ultrapure LPS from E. coli K12 (InvivoGen, Cat # tlrl-peklps) or 1 μg/mL synthetic triacylated lipopeptide Pam3CSK4 (InvivoGen, Cat # tlrl-pms) for 4 h and treated with 5 μM Nigericin sodium salt (AdipoGen, Cat # AG-CN2-0020-M005) or 5 mM Adenosine 5'-Triphosphate (ATP) disodium salt (Merck Life Science, Cat # 1191-1GM) for 2 h. To activate NLRP1, cells were primed and treated with 10 μM Talabostat mesylate (R & D Systems, Cat # 3719/10) or 10 μM anisomycin (Merck, Cat # A9789) for 24 h. To activate NAIP/NLRC4, cells were primed and treated with Lfn-BsaL (1 ng/mL, a fusion protein of the N-terminal domain of the Bacillus anthracis lethal factor and BsaL needle protein from *Burkholderia pseudomallei*, InvivoGen, Cat # tlrl-ndl) plus 250 ng/mL Anthrax Protective Antigen (PA), Recombinant from *Bacillus anthracis* (Quadratech Diagnostics, Cat # LL-171E) for 2 h. To activate the non-canonical inflammasome, iMacs were primed overnight with 1 μg/mL Pam3CSK4 (16 h) then fresh media was added and 10 μg/mL LPS was transfected using Lipofectamine LTX Reagent with PLUS Reagent (ThermoFisher, Cat # A12621) and centrifuged at 1000 G for 10 min at room temperature before being incubated for 5 h. To inhibit NLRP3, cells were pre-treated with 0.1–10 μM MCC950 (Caltag Medsystems, Cat # AG-CR1-3615-M005) for 30 min prior to treatment with nigericin or transfection with LPS. To inhibit caspase-1, cells were pre-treated with 20 μM VX-765 (MedChemExpress, Cat # HY-13205 -5 mg) for 30 min.

Flow cytometry

iMacs and HMDMs were resuspended and 0.5×10^6 added per flow tube in 1 mL DPBS. All cells were stained with eBioscience Fixable Viability Dye eFluor 506 (ThermoFisher, Cat # 65-0866-14) at a dilution of 1:500 for 10 min at room temperature then washed twice in DPBS. Blocking solution (Human TruStain FXX, Biolegend, Cat # 422302) was added at a dilution of 1:20 for 30 min in the dark at room temperature and cells were washed twice in DPBS. For intracellular antibodies, cells were resuspended in 200 μL fixation buffer (eBioscience Intracellular Fixation & Permeabilization Buffer Set, ThermoFisher, Cat # 88-8824-00) for 30 min at room temperature. The cells were centrifuged for 3 min at 800 G and resuspended in 200 μL permeabilization buffer (1:10 from the 10× stock, diluted in ddH20, eBioscience Intracellular Fixation & Permeabilization Buffer Set, ThermoFisher, Cat # 88-8824-00). The cells were centrifuged for 5 min at 500 G and the cell pellet resuspended in 500 μL permeabilization buffer. The appropriate antibody/isotype control concentration was added, and the cells were incubated for 30 min in the dark at 4°C. For extracellular antibodies, 500 μL DPBS was added to each tube with the appropriate antibody/isotype concentration and incubated for 30 min in the dark at 4°C. Cells were stained with antibodies and isotype controls listed in the key resources table. The flow tubes were centrifuged for 5 min at 500 G, room temperature and washed twice with DPBS. Cells stained for extracellular antibodies were resuspended in DPBS and cells stained for intracellular antibodies were resuspended in permeabilization buffer. Samples were run on the FACS Canto II cytometer (Becton Dickenson, UK) and results analyzed using FlowJo software (BD Biosciences, UK).

Phagocytosis assays

iMacs and HMDMs were plated at 1×10^5 per well in 24 well plates on coverslips in iMac medium and incubated overnight. RPMI was removed and replaced with OptiMEM (ThermoFisher, Cat # 31985070). Latex amine-modified polystyrene, fluorescent yellow-green beads (Sigma-Aldrich. Cat #L1030-1ML) were diluted 1:10 in DPBS and added 1:50 per well. iMacs were incubated with the fluorescent beads for 30 or 90 min. OptiMEM was removed and cells were washed in DPBS. Fresh OptiMEM was added and 0.5 μM LysoTracker red DND-99 (ThermoFisher, Cat #L7528) added for 30 min.

Upon completion of the experiment, cells were washed in DPBS and fixed in 4% paraformaldehyde (PFA) for 15 min. PFA was aspirated and coverslips were stored in PBS at 4°C until staining.

Coverslips were washed 3 times in PBS then removed from the 24 well plates and kept in a humidified chamber. Coverslips were permeabilized with 0.1% Triton X-100, washed in PBS and PFA was quenched with 50 mM ammonium chloride. Coverslips were washed then blocked with 0.5% BSA/PBS for 30 min before mounting in mounting media containing DAPI on glass microscope slides. The mounted coverslips were sealed with clear nail polish and stored at 4°C until imaging. Coverslips were imaged at 63 × (oil lens) using the Stellaris-5 confocal microscope. An overview of each coverslip was taken using the automated spiral scan on the Leica LAS X software. Images were taken at a 16-bit resolution using a 1024 × 1024 scan format. Representative images were analyzed using Fiji. The percentage of DAPI+bead+ cells was calculated.

Mass spectrometry

iMacs were plated at 1×10^6 in 6 well plates and challenged with LPS (100 ng/mL) for 6 or 24 h. HMDMs were plated 1×10^6 in 6 well plates and challenged with LPS (100 ng/mL) for 2 or 16 h. The cells collected in ice-cold PBS into Eppendorfs and centrifuged at 13,000 G at 4°C for 10 min. The cell pellets were frozen at −80°C. The cells were lysed with 1% SDC lysis buffer (1% SDC, 10 mM TCEP, 40 mM 2-chloroacetamide (CAA), 100 mM Tris pH 8.5) and heated for 10 min at 95°C, with shaking (800 RPM). Samples were sonicated for 2 min then centrifuged at maximum speed for 15 min and the supernatants stored at −20°C.

Preparation of iMac samples for mass spectrometry was carried out using a protein aggregation capture (PAC) protocol adapted from Kverneland et al.[52] using the Opentrons OT-2 liquid handling system. Lysates were added to MagReSyn Hydroxyl beads (ReSyn Biosciences) for aggregation for 20 min, with a mixing step at 10 min. The bead–protein aggregates were washed twice with

Acetonitrile (ACN) and once with 50 mM triethylammonium bicarbonate buffer (TEAB) pH 8.5 at a slow speed. Proteins were digested with 50 mM TEAB containing Lys-C and Trypsin at an enzyme-to-substrate ratio of 1:100 and 1:50, respectively for 4 h at room temperature. Evotips (Evosep) were activated by soaking in isopropanol for 10 s, then pre-conditioned by pushing through 20 μL of 0.1% FA. 50% of the peptides were loaded onto Evotips and mass spectrometry was performed on a timsTOF HT (Bruker) quadrupole time-of-flight mass spectrometer integrating trapped Ion Mobility (IM) separations coupled online via a Captivespray electrospray source (Bruker) to Evosep One liquid chromatography system.[53,54] Peptides were separated on an integrated packed emitter column (15 cm × 75 μm ID – Coann) packed with 1.5 μm Reprosil Saphir beads (Dr. Maisch) using whisper 40 SPD method. Data was acquired using a diaPASEF method consisting of 12 cycles including a total of 24 windows, covering m/z from 300 to 1400 Da with two mobility windows each, covering the ion mobility range (1/K0) from 0.70 to 1.42 V s/cm2. The CaptiveSpray source was kept at a capillary voltage of 1,600 V, with a dry gas flow rate of 3.0 L/min at 180°C. Accumulation and ramp times were set to 100 ms, resulting in 180 ms per MS^1 or MS^2 (DIA-PASEF) scan, excluding transfer time. The collision energy was programmed as a function of ion mobility, following a straight line from 20 eV for 1/K0 of 0.6 V s/cm2 to 59 eV for 1/K0 of 1.6 V s/cm2. To calibrate TIMS, the elution voltage was set to linear mode to obtain 1/K0 ratios using three ions (m/z of 622, 922, 1222) from the ESI-L Tuning Mix (Agilent). An additional ion-mobility gas phase fractionation run (IM-GPF) of a pooled sample was included to boost library coverage.[55]

HMDM samples were digested overnight on a shaking heat block at 37°C using MS-grade LysC and Trypsin protease. Sample processing was performed using an in-StageTip method adapted from Kulak et al.[56] Cartridges were made by punching out two small (2 mm diameter) disks of Styrene divinylbenzene reversed phase sulfonate (SDB-RPS) solid phase extraction disk (Empore #66886-U) and putting these carefully into p200 tips with the ends cut off. The tip was inserted into the adaptor (cut p1000 tip) on top of a 2 mL sterile Eppendorf. Cartridges were conditioned by washing with 100% acetonitrile and centrifuging for 3 min at 3000G. This was followed 2 washes 0.1% TFA in diH2O centrifuged for 3 min at 3000G. Samples were loaded into the cartridges and centrifuged twice, adding the sample flowthrough back in each time (2000G for 5 min). 1% TFA in isopropanol was added and the samples were centrifuged for 6 min at 2000G. This was repeated four times, discarding the flowthrough every two washes. diH2O with 0.1% TFA was added and centrifuged for 6 min at 2000G, twice, discarding flowthrough. Finally, peptides were eluted in elution buffer (80% ACN, 1% ammonia, in diH2O) and centrifuged for 5 min at 2000G. Eluate was vacuum centrifuged to dryness (Genevac MiVac Centrifugal Concentrator, OH mode, 45°C for 120 min). Peptides were resuspended in Buffer A (0.1% formic acid in water) prior to LC-MS acquisition.

Separation was performed on a nanoElute liquid chromatography system (Bruker), using the following gradient: 0 min, 3% Buffer B; 40 min 15% Buffer B; 65 min 25% Buffer B; 71 min 33% Buffer B; 77 min, 95% Buffer B; 82 min 95% Buffer B (flow rate 0.25μL/min). separation was performed on an integrated emitter column (25 cm × 75 μm) packed with 1.5 μm Reprosil Saphir beads. This was coupled online via a Captivespray electrospray source (Bruker) to a timsTOF Pro quadrupole time-of-flight mass spectrometer with trapped ion mobility separation (Bruker) operated in DIA mode, using a standard diaPASEF acquisition scheme.[53] Again, a representative sample was acquired using an IM-GPF acquisition scheme or library generation.[55]

Mass spectrometry data for iMac samples was analyzed in Spectronaut version 19.1 (Biognosys) using the using a direct DIA workflow with default BGS settings against UniProtKB human sequence database UP000005640 (downloaded January 2024 - 20,413 entries); specific Trypsin/P digestion, 2 missed cleavages, peptide length 7–52, carbamidomethyl cysteine set as fixed modification, oxidation of methionine and N-terminal methionine excision as variable modifications allowing up to five variable sites per peptide. For PSMs, peptides, and proteins, the false discovery rate was controlled at 1%. Precursor filtering required a peptide–spectrum match q-value of ≤0.01 and a minimum posterior error probability of 0.2. Protein identifications met q-value thresholds of 0.01 for experiment-wide assessment and 0.05 for run-wide assessment. Protein grouping was performed using the IDPicker algorithm. Protein quantification was performed using the MaxLFQ algorithm on peak areas, followed by cross-run normalization, without imputation of missing values. UniProtKB human sequence database UP000005640 (83,385 entries). HMDM data was analyzed in Spectronaut version 18.7 (Biognosys). A spectral library was generated, incorporating IM-GPF runs, using default BGS settings as before. Likewise, DIA analysis was performed using default BGS settings as before. The resulting protein quantities tables were exported using a pivot report in Spectronaut. Downstream plots were generated in R, using base R and the following packages: ggplot2, ggrepel, pheatmap. Heatmap clustering was performed using Euclidean distance and protein quantities are scaled by row.

Lactate dehydrogenase (LDH) assay

LDH assay (Cytotoxicity Detection Kit Roche, Sigma Aldrich, Cat # 11644793001) was performed following manufacturer's instructions to assess cell death.

Enzyme-linked immunosorbent assay (ELISA)

Human IL-1 beta/IL-1F2 DuoSet ELISA (R&D Systems, Cat# DY201), Human TNF-alpha DuoSet ELISA (R&D Systems, Cat# DY210) and human IL-18 ELISA (ThermoFisher, Cat# BMS267-2MST) were performed following manufacturer's instructions.

Western blot

Cell lysates were harvested in 50 μL cell lysis buffer (66 mM Tris HCl pH 7.4% SDS) with 1:10,000 BaseMuncher (Expedeon Cat # BM0025). 1× NuPAGE LDS sample buffer (Invitrogen, Cat # NP0007) plus Dithiothreitol (DTT, Merck Life Science, Cat # 20–265) was added to each sample. Samples were boiled for 5 min at 95°C then run on 4–20% Mini-PROTEAN TGX Precast Protein Gels

(BioRad, 10 wells, Cat #4561093, 12-wells, Cat # 4561095, or 15-wells, Cat # 4561096) and were separated for 60–80 min at 120 V alongside Precision Plus Protein Kaleidoscope Standard (BioRad, Cat # 1610375EDU). Proteins were transferred to a nitrocellulose membrane using the Trans-Blot Turbo Transfer System (BioRad) at 25V for 7 min. Membranes were blocked in 5% dried milk powder in TBS-Tween at room temperature for 1 h. Membranes were incubated with one of the following antibodies: caspase-1 (Cell Signaling Technology, Cat# 3866), cleaved caspase-1 (Cell Signaling Technology, Cat# 4199S), caspase-4 (Cell Signaling, Cat# 4450S), cleaved GSDMD (Abcam, Cat# ab215203), C-terminal GSDMD (Abcam, Cat# ab210070), NLRP3 (Cell Signaling, Cat# 15101), IL-1β (R&D Systems, Cat# AF401-NA), or β-actin (Sigma, Cat# A2228) overnight on a rocker 4°C. After incubation, the membranes were washed and incubated with the appropriate secondary antibody: anti-rabbit (Jackson Immunoresearch, Cat# 111- 035-003), anti-mouse (Jackson Immunoresearch, Cat# 515- 035-003), or anti-goat (Jackson Immunoresearch, Cat# 705- 035–147). Proteins were detected by incubating membranes with Clarity ECL (Biorad, Cat # 170–5061) and the fluorescence imaged using the GeneSys imager.

ASC speck staining and imaging

Cells were plated at 1×10^5 per well in their culture medium overnight in 24 well plates with coverslips added. Medium was then replaced with OptiMEM per well. The indicated stimuli were added for times listed then cells were washed in PBS and fixed in 4% paraformaldehyde (PFA) for 15 min. PFA was aspirated and coverslips were stored in PBS at 4°C until staining.

Coverslips were washed 3 times in PBS then removed from the 24 well plates and kept in a humidified chamber. Coverslips were permeabilized with 0.1% Triton X-100, washed in PBS and then PFA was quenched with 50 mM ammonium chloride. Coverslips were washed then blocked with 0.5% BSA/PBS for 30 min. Primary anti-ASC antibody (BioLegend, Cat # 676502) was diluted 1:50 in 0.5% BSA/PBS and added for 60 min in the dark then coverslips were washed in PBS 4 times for 5 min. Alexa Fluor 488 Donkey anti-mouse (Invitrogen, Cat # A21202) was diluted 1:800 in 0.5% BSA/PBS and added for 60 min in the dark. The coverslips were washed in PBS 4 times for 5 min before mounting in mounting media containing DAPI on glass microscope slides. The mounted coverslips were sealed with clear nail polish and stored at 4°C until imaging. Coverslips were imaged using the Leica LAS X software and the DM5500 fluorescent microscope at 20× and 40×. ASC specks were quantified using Fiji and the percentage of DAPI+ASC+ positive cells was calculated.

Polymorphism detection in RNA sequencing databases

Kolf_2 and ieki_2 iPSC RNA sequencing databases were accessed from https://www.hipsci.org/#/lines/H and BAM/BAI files analyzed using the Interactive Genome Viewer (IGV) human genome. The P2RX7 gene sequences were compared between the two cell lines and mismatches, insertions, and deletions were highlighted.

QUANTIFICATION AND STATISTICAL ANALYSIS

N = the number of kolf_2 or ieki_2 iMac replicates from one differentiation batch unless otherwise indicated. For datasets comparing the means of three or more groups with one independent variable, statistical significance was checked through performing a One-Way ANOVA with Tukey's post-test. For datasets comparing the means of three or more groups with more than one independent variable, statistical significance was checked through performing a two-Way ANOVA with Sidak's post-test. A power calculation was not performed. Graphs were generated using GraphPad Prism 9. Data presented as mean ± SEM, n number indicated in figure legends; n.s. not significant, *$p < 0.05$, **$p < 0.01$, ***$p < 0.001$, ****$p < 0.0001$. Specific statistical tests used are described in detail in figure legends.

Cell Reports Methods, Volume *6*

Supplemental information

Induced pluripotent stem cell-derived macrophages enable broad modeling of human inflammasome signaling

Chloe M. McKee, Melanie Cranston, Emma C. McKay, Mohammad Arefian, Thea J. Mawhinney, Ben C. Collins, and Rebecca C. Coll

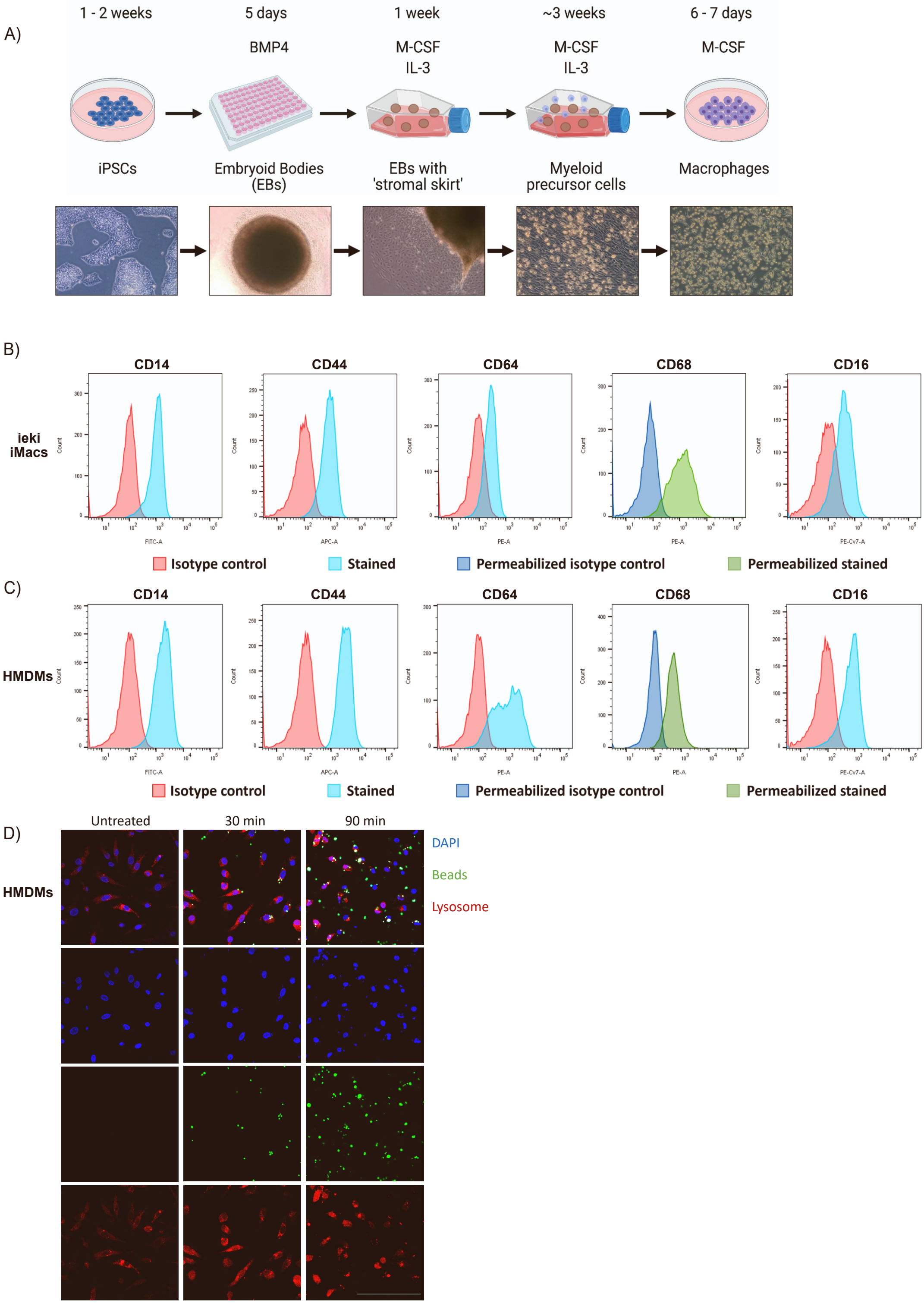
A)
1 - 2 weeks
5 days
1 week
~3 weeks
6 - 7 days
BMP4
M-CSF
IL-3
M-CSF
IL-3
M-CSF
iPSCs
Embryoid Bodies (EBs)
EBs with 'stromal skirt'
Myeloid precursor cells
Macrophages
B)
CD14
CD44
CD64
CD68
CD16
ieki iMacs
Isotype control
Stained
Permeabilized isotype control
Permeabilized stained
C)
CD14
CD44
CD64
CD68
CD16
HMDMs
Isotype control
Stained
Permeabilized isotype control
Permeabilized stained
D)
Untreated
30 min
90 min
HMDMs
DAPI
Beads
Lysosome

Fig. S1 HMDMs express macrophage markers and are highly phagocytic, related to Fig. 1.

A) Diagram illustrating the iMac differentiation protocol. Expression of macrophage markers in **B)** ieki_2 iMacs and **C)** HMDMs compared to relative isotype controls. **D)** Confocal microscope images from HMDMs following incubation with fluorescent beads. Scale bar = 100 µM.

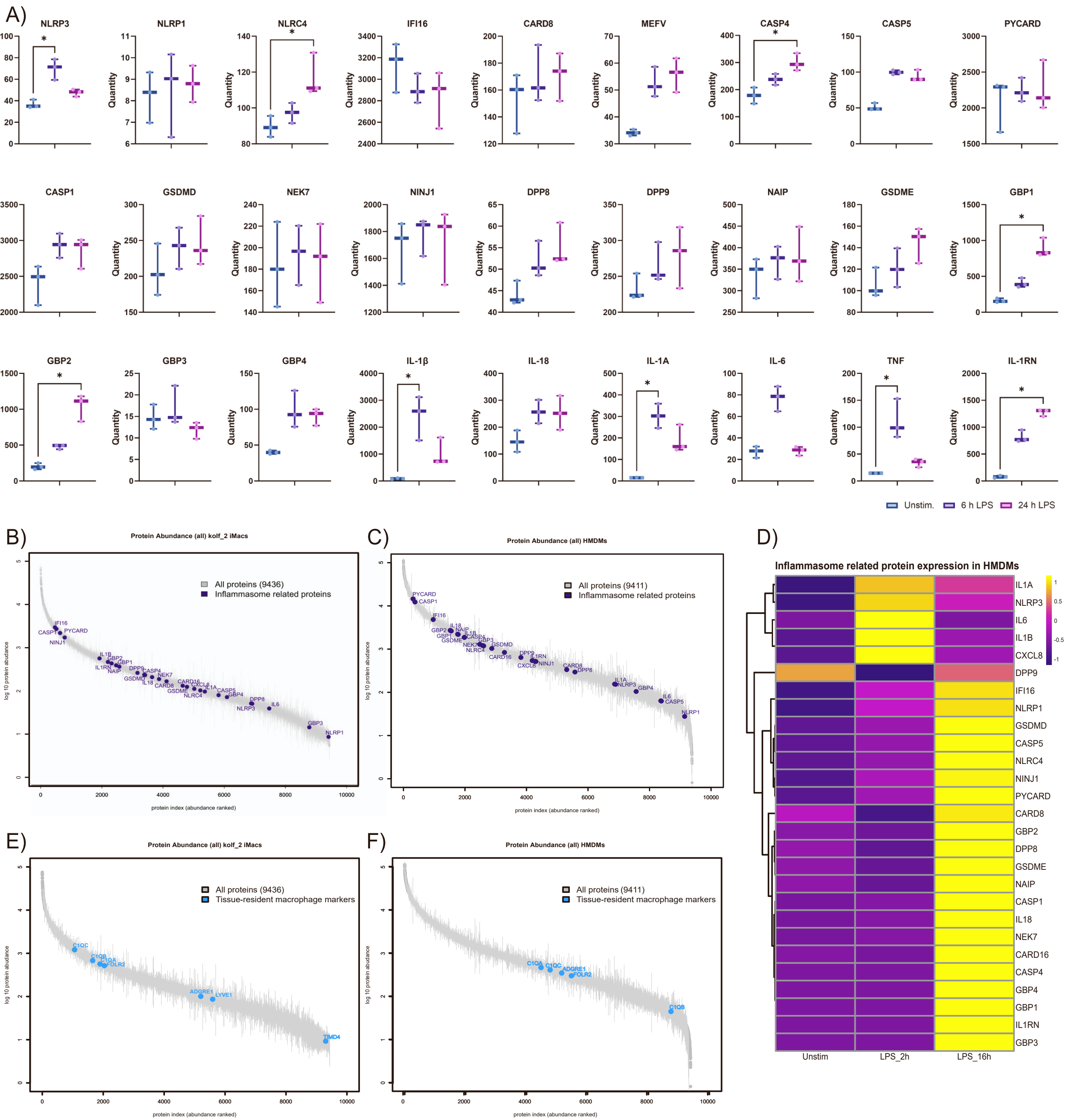
A)
NLRP3
NLRP1
NLRC4
IFI16
CARD8
MEFV
CASP4
CASP5
PYCARD
CASP1
GSDMD
NEK7
NINJ1
DPP8
DPP9
NAIP
GSDME
GBP1
GBP2
GBP3
GBP4
IL-1β
IL-18
IL-1A
IL-6
TNF
IL-1RN
Quantity
Unstim.
6 h LPS
24 h LPS
B)
Protein Abundance (all) kolf_2 iMacs
All proteins (9436)
Inflammasome related proteins
log 10 protein abudance
protein index (abundance ranked)
C)
Protein Abundance (all) HMDMs
All proteins (9411)
Inflammasome related proteins
log 10 protein abudance
protein index (abundance ranked)
D)
Inflammasome related protein expression in HMDMs
IL1A
NLRP3
IL6
IL1B
CXCL8
DPP9
IFI16
NLRP1
GSDMD
CASP5
NLRC4
NINJ1
PYCARD
CARD8
GBP2
DPP8
GSDME
NAIP
CASP1
IL18
NEK7
CARD16
CASP4
GBP4
GBP1
IL1RN
GBP3
Unstim
LPS_2h
LPS_16h
E)
Protein Abundance (all) kolf_2 iMacs
All proteins (9436)
Tissue-resident macrophage markers
C1QC
C1QB
C1QA
FOLR2
ADGRE1
LYVE1
TIMD4
log 10 protein abudance
protein index (abundance ranked)
F)
Protein Abundance (all) HMDMs
All proteins (9411)
Tissue-resident macrophage markers
C1QA
C1QC
ADGRE1
FOLR2
C1QB
log 10 protein abudance
protein index (abundance ranked)

Fig. S2 Expression of inflammasome-related proteins is similar between iMacs and HMDMs, related to Fig. 1. A) Expression of inflammasome- and inflammation-related proteins in kolf_2 iMacs following 6h/24h LPS. Data represented as mean +/- SEM, n = 3 replicates, *$p < 0.05$ by Two-way ANOVA with Šídák's multiple comparisons. Ranked average Log10 protein abundance of inflammasome-related proteins indicated in **B)** kolf_2 iMacs **C)** HMDMs. **D)** Protein quantity heatmap of inflammasome- and inflammation-related proteins following global proteomics analysis of HMDMs. Ranked average Log10 protein abundance of tissue-resident macrophage-associated proteins in **E)** kolf_2 iMacs and **F)** HMDMs. n = 3 kolf_2 iMac replicates, n = 4 buffy coat donors.

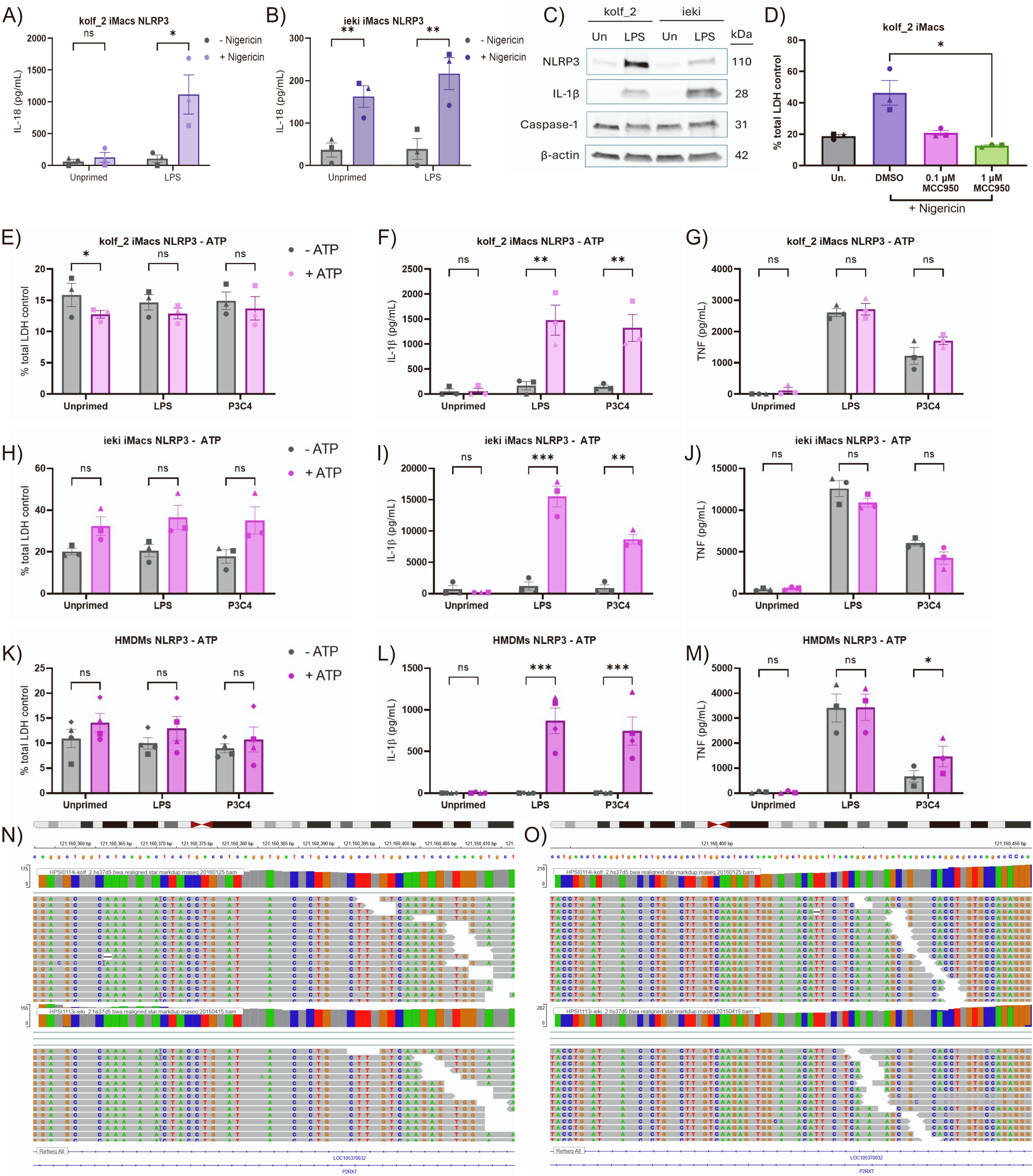

A)
kolf_2 iMacs NLRP3
IL-18 (pg/mL)
- Nigericin
+ Nigericin
ns
*
Unprimed
LPS
B)
ieki iMacs NLRP3
IL-18 (pg/mL)
**
**
Unprimed
LPS
C)
kolf_2
ieki
Un
LPS
kDa
NLRP3
110
IL-1β
28
Caspase-1
31
β-actin
42
D)
kolf_2 iMacs
% total LDH control
*
Un.
DMSO
0.1 µM MCC950
1 µM MCC950
+ Nigericin
E)
kolf_2 iMacs NLRP3 - ATP
% total LDH control
- ATP
+ ATP
Unprimed
LPS
P3C4
F)
kolf_2 iMacs NLRP3 - ATP
IL-1β (pg/mL)
G)
kolf_2 iMacs NLRP3 - ATP
TNF (pg/mL)
H)
ieki iMacs NLRP3 - ATP
% total LDH control
I)
ieki iMacs NLRP3 - ATP
IL-1β (pg/mL)
J)
ieki iMacs NLRP3 - ATP
TNF (pg/mL)
K)
HMDMs NLRP3 - ATP
% total LDH control
L)
HMDMs NLRP3 - ATP
IL-1β (pg/mL)
M)
HMDMs NLRP3 - ATP
TNF (pg/mL)
N)
O)
Refseq All
LOC105370032
P2RX7

Fig. S3 NLRP3 activation in response to ATP is broadly comparable in iMacs and HMDMs, related to Fig. 2. IL-18 release from **A)** kolf_2 and **B)** ieki_2 iMacs following 4h LPS and 2h nigericin. **C)** Western blot from unstimulated and 6h LPS-primed kolf_2 and ieki_2 iMacs. **D)** LDH release from kolf_2 iMacs pre-treated with MCC950 (30 min) before nigericin (2h). LDH, IL-1β, and TNF release from **E-G)** kolf_2 iMacs, **H-J)** ieki_2 iMacs, and **K-M)** HMDMs following 4h LPS/Pam3CSK4 and 2h ATP. Data represented as mean +/- SEM, n = 3 iMac replicates, n = 4 buffy coat donors, *$p < 0.05$, **$p < 0.01$, ***$p < 0.001$, ****$p < 0.0001$ by Two-way ANOVA with Šídák's multiple comparisons. **N)** and **O)** Representative IGV screenshots showing read alignments (BAM files) for kolf_2 iPSCs (top) and ieki_2 iPSCs (bottom) indicating mismatches (transparent), insertions (lilac), and deletions (-) between the two cell lines.

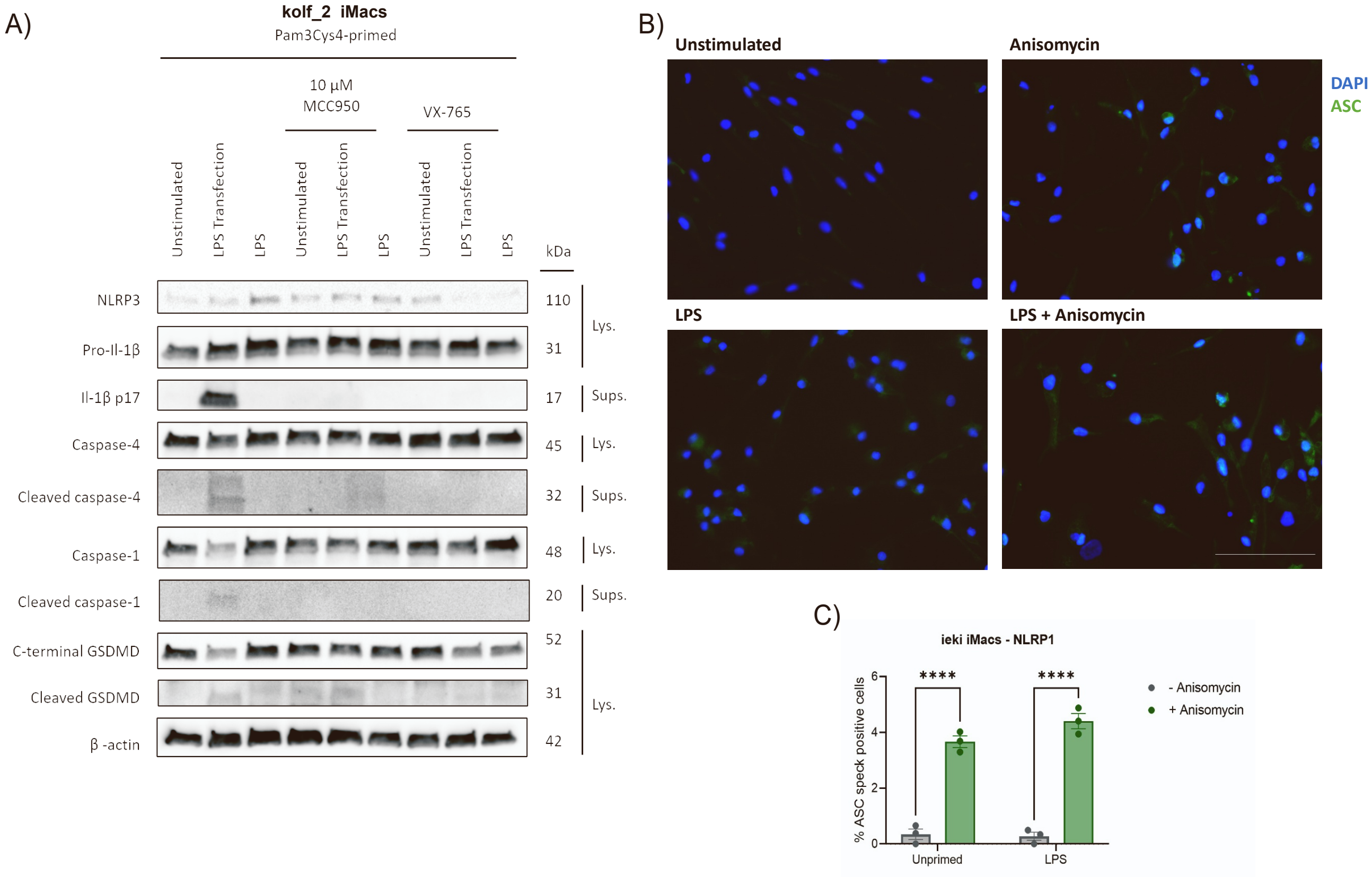

Fig. S4 NCI activation and NLRP1-induced ASC speck formation in iMacs, related to Fig. 3 and Fig. 4. A) Western blot from 16h Pam3CSK4-primed kolf_2 iMacs following 5h LPS transfection or LPS stimulation and pre-treatment with MCC950 or VX-765 (30 min). **B)** Fluorescent images from ieki_2 iMacs following 4h LPS and 24h anisomycin. Scale bar = 100 µM. **C)** Quantification of ASC speck positive cells. Data represented as mean +/- SEM, n = 3 iMac ieki_2 replicates, *p< 0.05, ***p< 0.001, ****p< 0.0001 by repeated measures ANOVA with Tukey's multiple comparisons test.

Donor	Date	Blood Type	Sex	Haemochromatosis (Y/N)	Figure No
1	27.10.20	N/A	N/A	N/A	4D-F
2	10.11.20	N/A	N/A	N/A	4D-F
3	26.11.20	N/A	N/A	N/A	4D-F
4	13.01.21	N/A	N/A	N/A	4D-F
5	26.01.21	N/A	N/A	N/A	4D-F
6	02.06.21	N/A	N/A	N/A	2 G-I, S4K-M
7	15.06.21	N/A	N/A	N/A	2 G-I, S4K-M
8	20.07.21	N/A	N/A	N/A	2 G-I, S4K-M
9	03.08.21	N/A	N/A	N/A	2 G-I, S4K-M
10	13.01.23	B+	N/A	N	S3B,C,E
11	13.01.23	O-	N/A	N	S3B,C,E
12	20.01.23	O+	N/A	N	S3B,C,E
13	20.01.23	O+	N/A	N	S3B,C,E
25	01.02.24	O-	F	N	1C, 3I-K, S1B
26	01.02.24	A	F	N	1C & F, 3I-K, S1B & D
27	15.02.24	O+	F	N	1C, 3I-K, S1B
28	15.02.24	A+	F	N	1C, 3I-K, S1B
31	22.03.25	O+	M	N	1F, S1D
32	22.03.25	O+	F	N	1F, S1D
39	29.07.24	A-	F	N	3D, E
49	17.1.25	A-	M	N	3D, E
50	17.1.25	A+	F	N	3D, E
51	27.11.25	A+	F	N	4J-L
52	05.02.26	O-	F	N	4J-L
53	05.02.26	B+	F	N	4J-L

Table S2 Buffy coat characteristics, related to STAR Methods.
